# Integrating Cacao Physicochemical-Sensory Profiles via Gaussian Processes Crowd Learning and Localized Annotator Trustworthiness

**DOI:** 10.3390/foods14172961

**Published:** 2025-08-25

**Authors:** Juan Camilo Lugo-Rojas, Maria José Chica-Morales, Sergio Leonardo Florez-González, Andrés Marino Álvarez-Meza, German Castellanos-Dominguez

**Affiliations:** 1 Signal Processing and Recognition Group, Universidad Nacional de Colombia, Manizales 170003, Colombia; amalvarezme@unal.edu.co (A.M.Á.-M.); cgcastellanosd@unal.edu.co (G.C.-D.); 2 Casa Luker, Calle 13 # 68-98, Zona Industrial, Bogotá 110901, Colombia; mchica@lukerchocolate.com (M.J.C.-M.); sflorez@lukerchocolate.com (S.L.F.-G.)

**Keywords:** crowd learning, Gaussian processes, annotator modeling, sensory evaluation, cacao

## Abstract

Understanding the intricate relationship between sensory perception and physicochemical properties of cacao-based products is crucial for advancing quality control and driving product innovation. However, effectively integrating these heterogeneous data sources poses a significant challenge, particularly when sensory evaluations are derived from low-quality, subjective, and often inconsistent annotations provided by multiple experts. We propose a comprehensive framework that leverages a correlated chained Gaussian processes model for learning from crowds, termed MAR-CCGP, specifically designed for a customized Casa Luker database that integrates sensory and physicochemical data on cacao-based products. By formulating sensory evaluations as regression tasks, our approach enables the estimation of continuous perceptual scores from physicochemical inputs, while concurrently inferring the latent, input-dependent reliability of each annotator. To address the inherent noise, subjectivity, and non-stationarity in expert-generated sensory data, we introduce a three-stage methodology: (i) construction of an integrated database that unifies physicochemical parameters with corresponding sensory descriptors; (ii) application of a MAR-CCGP model to infer the underlying ground truth from noisy, crowd-sourced, and non-stationary sensory annotations; and (iii) development of a novel localized expert trustworthiness approach, also based on MAR-CCGP, which dynamically adjusts for variations in annotator consistency across the input space. Our approach provides a robust, interpretable, and scalable solution for learning from heterogeneous and noisy sensory data, establishing a principled foundation for advancing data-driven sensory analysis and product optimization in the food science domain. We validate the effectiveness of our method through a series of experiments on both semi-synthetic data and a novel real-world dataset developed in collaboration with Casa Luker, which integrates sensory evaluations with detailed physicochemical profiles of cacao-based products. Compared to state-of-the-art learning-from-crowds baselines, our framework consistently achieves superior predictive performance and more precise annotator reliability estimation, demonstrating its efficacy in multi-annotator regression settings. Of note, our unique combination of a novel database, robust noisy-data regression, and input-dependent trust scoring sets MAR-CCGP apart from existing approaches.

## 1. Introduction

Cocoa-based products carry profound cultural significance, offer essential nutritional value, and underpin a multibillion-dollar global industry that supports the livelihoods of millions in rural communities [1]. Given that consumer acceptance is largely driven by the perception of sensory attributes such as aroma, flavor, and texture, comprehensive sensory profiling has become critical, not only for quality assurance and regulatory compliance but also for the preservation of geographical indication and origin certification schemes [2,3,4]. However, the human evaluations that form the basis of these sensory assessments are intrinsically subjective, with panelist perceptions varying according to factors such as experience, fatigue, and environmental conditions during tasting [5,6].

To address this inherent variability, researchers increasingly integrate diverse instrumental measurements with advanced chemometric and machine learning approaches. Techniques such as ultra-high-performance liquid chromatography and high-resolution mass spectrometry-based sensomics enable the identification of molecular determinants linked to both sensory quality and geographic origin [7]. In parallel, multivariate statistical tools like Orthogonal Partial Least Squares Discriminant Analysis (OPLS-DA) have proven effective in associating physicochemical variables—such as pH, °Brix, and polyphenol content—with specific flavor descriptors [8]. High-throughput metabolomics workflows, including platforms like FlavorMiner, facilitate the extraction of latent flavor signatures from complex, large-scale datasets [9]. Moreover, mixed-effects modeling frameworks have been employed to explicitly account for panelist-level noise and inter-individual variability during critical post-harvest stages such as fermentation and drying [10].

Nevertheless, sensory datasets often remain limited in size and characterized by high levels of noise, primarily due to the substantial time and cost associated with assembling and maintaining trained sensory panels. Moreover, inter-annotator reliability tends to fluctuate depending on the intrinsic chemical complexity of the samples under evaluation [11,12,13,14,15]. Within this evolving methodological landscape, basic physicochemical parameters, such as pH, fat content, polyphenol concentration, and key volatile compounds, have consistently demonstrated strong and reproducible correlations with sensory attributes [6]. As a result, these indicators offer practical and reliable proxies for predicting cocoa quality, particularly in contexts where comprehensive sensory evaluation is unfeasible.

Modern machine learning increasingly addresses the challenges posed by low-quality datasets—characterized by limited size, noise, sparsity, and subjectivity—by employing robust, noise-tolerant models and integrative frameworks. One notable trend is the application of deep learning methods with built-in noise-resilience or regularization mechanisms designed to extract reliable patterns from small and unreliable datasets. These techniques often include ensemble learning, dropout, and variational inference methods. For instance, in the domain of metabolomics-driven prediction in vegetable foods, deep learning models have shown strong performance even when faced with noisy and missing data by effectively integrating heterogeneous sources [16]. In food science applications, the data quality issues are exacerbated by the use of sensor-based and crowd-sourced annotations, often yielding highly subjective and inconsistent datasets. Advanced methods leveraging spectroscopy and hyperspectral imaging integrated with Artificial Intelligence (AI) have been used to address such limitations. These methods include deep architectures that can model high-dimensional, noisy inputs effectively, as shown in grain adulteration detection and cereal quality assessment tasks [17]. Furthermore, food authentication technologies such as electronic nose and tongue systems have increasingly adopted machine learning strategies that not only enhance detection accuracy but also account for sensor noise and low signal-to-noise ratios in complex food matrices [18]. Also, systematic reviews underscore that robustness to overlapping classes, outliers, and subjective assessments is critical in these contexts, often requiring hybrid models or pre-processing stages for noise filtration [19]. These advancements are particularly relevant for sensory profiling applications, where subjective human input is prevalent.

In turn, recent advances in machine learning increasingly rely on crowd-sourced or multi-annotator datasets, especially in domains where expert labeling is prohibitively expensive or time-consuming [20,21]. Traditional approaches often adopt label aggregation methods—such as Majority Voting (MV) or Expectation-Maximization (EM)—to create a single consensus label from multiple annotations [22]. Still, these techniques assume homogeneous annotator reliability and independence, which rarely holds in real-world scenarios. To overcome these limitations, modern frameworks now model annotator behavior as a function of the input space, using probabilistic tools like Gaussian Processes (GPs) [23], or deep neural networks that learn both the task and annotator reliability simultaneously [24]. One prominent example is the Correlated Chained Gaussian Processes with Generalized Cross Entropy (CCGP-GCE), which jointly models label noise and inter-annotator dependencies while leveraging a robust loss function to mitigate the impact of outliers and adversarial annotators [20].

Likewise, a growing trend in crowd learning research is the estimation of annotator-specific trustworthiness that varies across the input domain. Unlike earlier models that assigned fixed reliability scores, recent methods estimate annotator accuracy dynamically, using latent variables and input-conditioned reliability functions [25]. For instance, CCGP-GCE incorporates sigmoid-transformed latent functions to model each annotator’s reliability in a Bayesian framework, enabling soft decisions between trustworthy and noisy labels at each instance [20]. This is particularly effective when combined with the Generalized Cross Entropy loss, which blends the robustness of mean absolute error (MAE) with the fast convergence of cross-entropy (CE) [26]. Nevertheless, key challenges persist: scalability is limited by the computational complexity of Gaussian Process inference, and the lack of ground truth hampers the evaluation of trustworthiness estimations. Moreover, deep learning models that disregard inter-annotator correlations often underperform compared to Bayesian alternatives that explicitly encode annotator dependencies. As such, state-of-the-art methods increasingly favor hybrid approaches that integrate probabilistic modeling, structured priors, and noise-robust objectives to learn from complex, inconsistent crowd-sourced data.

In particular, in regression tasks involving multiple annotators, labeler variability stems from a range of factors, including differing levels of expertise, perceptual biases, and context-dependent interpretations. Rather than treating this variability as random noise, recent studies suggest that it often reflects systematic patterns tied to task structure and data representation [27]. For example, traditional agreement metrics fall short in continuous signal domains, where finer-grained discrepancies among annotators are meaningful and can be predictive [27]. Automated methods have also been introduced to inspect and interpret such variability, revealing that annotator behavior itself may encode relevant information [28]. Furthermore, annotator consistency—commonly used as a proxy for reliability—is not constant across the input space. Instead, it varies with the characteristics of the data, underscoring its inherently non-stationary nature [29].

To address the pressing challenge of integrating heterogeneous and noisy sensory data with physicochemical profiles in food science, we propose a novel Multi-Annotator Regression framework based on Correlated Chained Gaussian Processes (MAR-CCGP). Unlike prior approaches, MAR-CCGP explicitly models input-dependent annotator trustworthiness while simultaneously estimating continuous perceptual scores from physicochemical inputs. This dual modeling capability is especially critical in sensory evaluation contexts, where annotations are sparse, subjective, and non-stationary. We outline the following key stages of our framework:

– Construction of the LUKER-CACAO database, aligning standardized physicochemical measurements with sensory annotations from multiple expert panelists.
– Application of the MAR-CCGP framework to learn latent ground truth sensory scores and context-dependent annotator reliability through a shared latent factor model from noisy multiple annotator regression data.
– Development of a localized annotator trust score, leveraging the model’s posterior distribution to assess reliability per annotator and per sample.

We conduct controlled experiments on both real-world and semi-synthetic datasets to benchmark performance and interpretability. Our experiments span the proprietary LUKER-CACAO dataset (five physicochemical inputs, and eight sensory descriptors rated by five annotators) and multiple semi-synthetic benchmarks derived from UCI regression datasets, where structured, region-specific noise profiles were simulated to emulate non-stationary annotator behavior. We benchmark MAR-CCGP against GPR-GT (GP on ground truth) [30], GPR-AVG (GP on consensus averages) [23], and LKAAR (a localized kernel alignment model for annotator relevance) [31]. Results demonstrate that MAR-CCGP consistently achieves superior predictive performance—often approaching the oracle—even in the absence of ground-truth labels. It recovers localized annotator trust profiles and outperforms consensus-only and partially localized models by capturing both inter-annotator correlations and input-conditioned noise structures. Overall, MAR-CCGP offers a suitable and interpretable solution for learning from subjective, sparse, and inconsistent annotations, with significant implications for robust modeling in food quality control.

Section 2 details the materials and methods employed in the study. Section 3 introduces the experimental setup. Section 4 presents and discusses the results. Finally, Section 5 provides the concluding remarks and future perspectives.

## 2. Materials and Methods

### 2.1. Casa Luker–Cacao Physicochemical-Sensory Dataset (LUKER-CACAO)

The dataset used in this study was developed in collaboration with Casa Luker (Luker Chocolate https://lukerchocolate.com/en/, accessed on 3 July 2025) and contains a collection of sensory and physicochemical measurements derived from cacao-based product evaluations. These data originate from routine quality control and research procedures applied to a wide variety of product types such as cocoa liquors, dark chocolate, milk chocolate, white chocolate, and cocoa powders.

The original dataset was compiled from two distinct sources: 15 quarterly technical reports containing physicochemical measurements, and 704 sensory evaluation session files. The physicochemical data were obtained through standardized instrumental analyses, while the sensory sessions involved trained panelists who provided evaluations of cacao production samples according to established sensory protocols. To construct a unified and analyzable dataset, all reports were systematically parsed using automated data extraction pipelines. The relevant tabular data were consolidated into two structured matrices: one representing the physicochemical descriptors (input features) and the other capturing the sensory evaluations (multi-annotator outputs). The sensory matrix adopts a structured format in which each row corresponds to a product sample and each column represents a specific sensory descriptor provided by an individual annotator, reflecting the inherent variability and sparsity of crowd-sourced annotations.

A subset of variables from both data modalities was subsequently selected to ensure consistency and relevance. For the physicochemical domain, five variables were retained based on their completeness across samples and their potential to capture key aspects of product structure and composition:

– *Moisture*: It refers to the amount of water present in a cocoa-based product, expressed as a percentage of the product’s total weight (%). Moisture content significantly impacts physical attributes such as texture, shelf-life, and microbial stability. More critically, it modulates the release and perception of flavor compounds during consumption. Variations in moisture influence the volatilization of aroma molecules and alter the way flavors are experienced in the mouth, thereby reshaping the sensory profile in terms of intensity, balance, and mouthfeel [32]. In the dataset, moisture values—reported on a dry-basis—range from 0 to approximately 200%.
– *Fat content*: It represents the proportion of lipids present in cocoa-based products, expressed as a percentage of total weight (%). Its modulation influences physical properties like viscosity, structure, and film formation capacity. These physical changes affect how the product interacts with the mouth during consumption, thereby altering the sensory profile by modifying lubrication, mouthfeel, and perception of flavor release [33]. In the dataset, values range from 0 to 60%.
– *Granulometry*: It measures the size and distribution of solid particles in cocoa-based products, expressed in micrometers (μm). Granulometry influences how particles interact and pack together, altering viscosity, flow behavior, and ultimately the perception of mouthfeel during consumption. Changes in granulometry reshape the sensory profile by modifying sensations like smoothness, thickness, and creaminess, which are critical for consumer acceptance [34]. Granulometry values range from 0 to 58 μm.
– *Plastic viscosity*: Measures the resistance of the product to flow after yielding has occurred, expressed in Pascal-seconds (Pa·s). It also reflects how easily the material continues to deform under applied shear during oral processing. Variations in plastic viscosity affect the sensory profile by altering the perceived thickness, smoothness, and creaminess during consumption. These changes shape the overall mouthfeel, influencing whether the product is experienced as rich, velvety, or fluid [34]. Observed values range from 0 to approximately 10.5 Pa·s.
– *Yield stress*: Represents the minimum force required to initiate flow in the product, expressed in Pascals (Pa). It is closely related to the structural integrity of the product before deformation starts. Variations in yield stress affect the sensory profile by altering initial mouthfeel sensations such as firmness and body, shaping the consumer’s perception of texture at the start of consumption [34]. Yield stress values range from 0 to approximately 62 Pa.

Likewise, from the sensory domain, eight attributes were selected based on their relevance to flavor perception and the density of available annotations. These attributes represent core dimensions of sensory quality. In trained descriptive analysis of cocoa-based products, sensory attributes are evaluated by expert panels using intensity scales ranging from 1 (absence) to 10 (maximum perceived intensity). These attributes capture key sensory dimensions affecting product quality, processing control, and consumer acceptance [35]:

– *Acidity*: It refers to the perception of sourness resulting from organic acids formed during fermentation. When present at appropriate levels, acidity can enhance brightness and complexity; however, excessive acidity is considered a defect, particularly in fine chocolate [36].
– *Bitterness*: It reflects the presence of alkaloids (primarily theobromine) and polyphenols, compounds inherent to cocoa. While some degree of bitterness is characteristic and desirable, excessive levels can disrupt sensory balance and negatively impact consumer acceptance [37].
– *Aroma*: It encompasses volatile compounds responsible for cocoa’s characteristic smells (fruity, floral, roasted), highly influenced by fermentation and roasting [32].
– *Astringency*: It refers to the drying, puckering sensation caused by interactions between polyphenols and salivary proteins. While moderate astringency can contribute positively to mouthfeel and complexity, excessive levels are perceived as unpleasant and may negatively impact sensory acceptance. [37].
– *Sweetness*: It reflects sugar content, critical to balancing bitterness and acidity for overall flavor harmony.
– *Hardness*: It describes the resistance during biting or deformation, influenced by fat content, tempering, and particle size.
– *Melting speed*: It reflects how quickly the product liquefies in the mouth, depending on fat composition and tempering. Faster melting generally enhances flavor release and mouthfeel [35].
– *Global impresssion*: It summarizes overall product quality, integrating flavor, aroma, and texture into a single judgment.

Of note, the analytical procedures used to obtain these physicochemical and sensory measurements are standardized and traceable to established methods. Table 1 summarizes the official protocols used for each selected measurement type.

**Figure 1:**
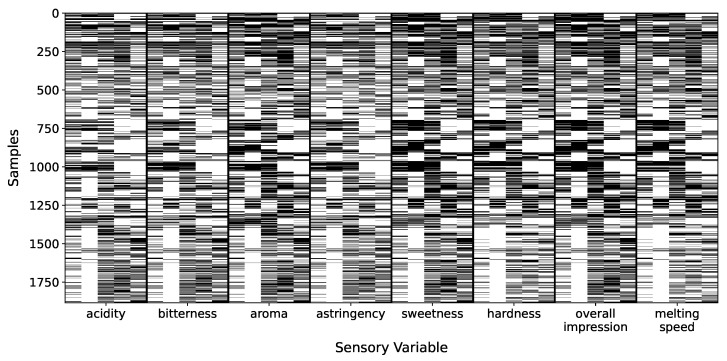
Annotation–coverage heatmap (black = rating present, white = missing) for the sensory-evaluation dataset. Each of the 1884 rows corresponds to a product sample; the 40 columns are grouped into the eight sensory variables—in order, *acidity*, *bitterness*, *aroma*, *astringency*, *sweetness*, *hardness*, *overall impression*, and *melting speed*. Within every block the same five panelists appear left-to-right (annotator IDs 135, 154, 155, 160, 179). Vertical grid lines thus mark variable boundaries, while the repeating column pattern allows per-annotator comparisons across variables. Horizontal white bands (e.g., in the *acidity* and *astringency* blocks) reveal samples that were never rated for that variable, whereas the sparse fourth column of several blocks highlights annotator 160’s systematically low participation—most pronounced on *bitterness* and *aroma*. The figure makes explicit both sample-level label sparsity and annotator-specific missingness patterns that motivate the MAR-CCGP modeling strategy.

**Figure 2:**
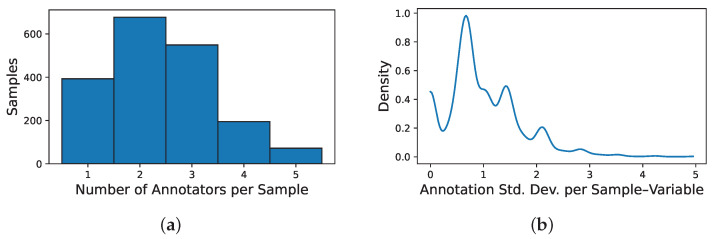
Inconsistencies in annotator behavior. (**a**) Number of annotators with at least one variable labeled. Shows, for each sample, how many annotators provided at least one label across the eight sensory attributes, revealing a typical coverage of two to three annotators per sample. (**b**) Distribution of standard deviation among annotator scores per sample–variable. Presents a kernel density estimate of the standard deviation in annotator scores for each sample–variable pair, quantifying how much annotators typically disagree. Most annotations exhibit standard deviations below 2.0, but a notable long tail indicates the presence of substantial inter-annotator variability in a subset of evaluations.

Next, to ensure adequate representation and consistency within the multi-annotator framework, the analysis was further restricted to a subset of five annotators, identified by the codes 135, 154, 155, 160, and 179. These annotators were selected based on their high annotation coverage across the chosen sensory attributes, enabling more reliable modeling of expert-specific patterns and behaviors. Namely, after merging we built an input–output, multi-annotator dataset with 1886 samples, five physicochemical features, and eight sensory outputs from five experts. Figure 1 presents the annotation coverage heatmap, grouping columns by sensory variable and ordering annotators within each block; black squares indicate present labels and white squares missing ones. This visualization highlights that certain variables include complete samples lacking annotations from any expert. Additionally, it reveals systematic patterns in annotator behavior—for instance, the fourth column in each block consistently shows fewer labels provided for attributes such as bitterness and aroma. Figure 2 further quantifies these patterns, providing a detailed overview of inconsistencies in annotator behavior: panel (a) shows the distribution of how many annotators labeled each sample—most receive labels from two to three experts—while panel (b) depicts the distribution of the standard deviation in annotator scores per sample–variable pair, revealing a concentration of low disagreement but also a long tail of cases with high annotator variability, emphasizing the challenge of modeling subjective sensory assessments.

Although we limited the present study to the five panelists with the highest annotation coverage, the MAR-CCGP framework is agnostic to the number of annotators and can readily accommodate larger or rotating sensory panels. Including additional experts who contribute only a few ratings would not hinder the model; rather, it would naturally lead to wider predictive intervals and more variable trust estimates for those sparsely represented individuals and for regions of the physicochemical space where data are scarce. Also, the method remains applicable to broader sensory panels, but its confidence in a particular annotator (or sample type) grows in proportion to the amount of information available.

For each selected sensory attribute, we constructed a dedicated dataset by intersecting the cleaned sensory and physicochemical records. The data were restricted to the previously selected subset of annotators and physicochemical features. To ensure that the learning models are trained on complete and reliable input–output pairs, we only retained samples that satisfied two conditions: (i) no more than one missing value among the physicochemical features, and (ii) no more than one missing annotation across the selected annotators for the target attribute.

This filtering strategy strikes a balance between strict completeness and reasonable sample retention, allowing us to preserve useful samples without compromising model integrity. Table 2 summarizes the percentage of available labels for each annotator across all sensory attributes. Table 3 shows the completeness of each physicochemical input feature, along with the final number of retained samples for each sensory regression task.

After applying the completeness constraints and constructing the final datasets for each sensory attribute, a final imputation step was performed to handle any remaining missing values in the physicochemical input variables. We employed the Iterative Imputer from scikit-learn, which estimates missing values by sequentially modeling each variable as a function of the others. This approach preserves inter-variable correlations and provides a flexible, data-driven completion method suitable for small datasets [43]. No imputation was applied to the sensory labels. Missing annotations were treated as unobserved and marginalized during training.

**Figure 3:**
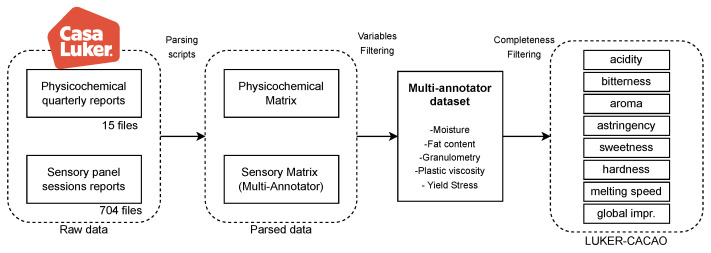
Pipeline for the construction of the LUKER-CACAO database.

The overall data curation workflow is summarized in Figure 3. This diagram outlines the end-to-end process from raw data acquisition through parsing, variable selection, and completeness filtering, culminating in the construction of the LUKER-CACAO dataset. It highlights the progressive reduction and refinement of data needed to obtain high-quality, multi-annotator datasets suitable for learning reliable mappings between physicochemical properties and sensory perceptions.

### 2.2. Correlated Chained Gaussian Processes (CCGPs)

Let a Gaussian Process (GP) be a collection of random variables f(x)∈R indexed by the input samples $x\in R^{P}$ such that any finite number of them follows a joint Gaussian distribution. Regarding this, a GP is completely specified by its mean $\mu_{x}=E\left\{f(x)\right\}$ ($\mu_{x}=0$ for simplicity) and covariance $\kappa \left(x,x^{\prime }\right)=E\left\{(f\left(x-\mu_{x}\right)\left(f\left(x^{\prime }\right)-\mu_{x^{\prime }}\right))\right\}$, where $\kappa_{f}:R^{P}\times R^{P}\to R$ is a kernel function, $x^{\prime }\in R^{P}$, and $E\left\{\cdot \right\}$ stands for the expectation operator [30]. Then: $f\left(x\right)∼\mathrm{GP}(0,\kappa \left(x,x^{\prime }\right))$.

Now, let $D=\{X\in R^{N\times P},y\in R^{N}\}$ denote an input–output dataset consisting of *N* samples and *P* input features, where X represents the input matrix and y is the corresponding vector of continuous regression targets. We define the latent function evaluations over the dataset as $f={\left[f\left(x_{1}\right),f\left(x_{2}\right),\ldots ,f\left(x_{N}\right)\right]}^{⊤}\in R^{N}$, where $f∼\mathrm{GP}(0,K_{ff})$, being $K_{ff}\in R^{N\times N}$ a kernel matrix computed by evaluating the covariance function $\kappa_{f}\left(x_{n},x_{n^{\prime }}\right)$ over all pairs of input points ($n,n^{\prime }=\left\{1,2,\ldots ,N\right\}$).

Remarkably, GPs inherit the properties of multivariate Gaussian distributions, allowing for linear combinations of latent functions and facilitating the construction of additive models. When coupled with an appropriate link function, this framework can be naturally extended to accommodate non-Gaussian observation models, as in generalized linear model settings [44]. Moreover, in the general case of GP-based models for supervised learning, the observed data D is modeled by constructing a joint distribution that combines a conditional likelihood with one or more latent functions governed by independent GP priors. If each parameter of the likelihood (e.g., noise variance, mean shift) is itself modeled as a latent function over the input space, the resulting structure is known as a Chained Gaussian Process (CGP) [45]. Formally, the joint distribution over y and *J* latent functions (LFs) $\hat{f}={\left[f_{1},\ldots ,f_{J}\right]}^{⊤}\in R^{NJ}$, conditioned on the input matrix X, yields:

(1) $$p\left(y,\hat{f}|X\right)=\prod_{n=1}^{N}p\left(y_{n}|\theta_{1}\left(x_{n}\right),\ldots ,\theta_{J}\left(x_{n}\right)\right)\prod_{j=1}^{J}N\left(f_{j}|0,K_{f_{j}f_{j}}\right),$$

where the likelihood parameters $\theta_{j}\left(x_{n}\right)$, with j∈{1,2,…,J}, are nonlinear mappings from GP priors, e.g., $\theta_{j}\left(x\right)=h_{j}\left(f_{j}\left(x\right)\right)$, with $h_{j}:R\to M_{j}$ mapping each $f_{j}\left(x\right)$ to the appropriate domain $M_{j}$. In addition, $f_{j}={\left[f_{j}\left(x_{1}\right),\ldots ,f_{j}\left(x_{N}\right)\right]}^{⊤}\in R^{N}$ is an LF vector that follows a GP prior and $K_{f_{j}f_{j}}\in R^{N\times N}$ is a kernel-based covariance matrix.

GPs, being nonparametric models, inherently scale poorly with dataset size due to their computational complexity of $O(N^{3})$ for a regression task defined over D. To address this limitation, we adopt a widely used approximation strategy that introduces a set of M≪N inducing variables, denoted by $u_{j}={\left[f_{j}\left(z_{1}^{j}\right),\ldots ,f_{j}\left(z_{M}^{j}\right)\right]}^{⊤}\in R^{M}$, where $Z_{j}=\left[z_{1}^{j},\ldots ,z_{M}^{j}\right]\in R^{P}$. This sparse approximation reduces the computational cost of inference and learning to $O(NM^{2})$. Under this formulation, the CGP prior yields [46]:

(2) $$p\left(f_{j},u_{j}\right)∼N\left(\left[\begin{array}{c} f_{j}\\ u_{j} \end{array}\right]|\;0,\left[\begin{array}{cc} K_{f_{j}f_{j}} & K_{f_{j}u_{j}}\\ K_{u_{j}f_{j}} & K_{u_{j}u_{j}} \end{array}\right]\right).$$

Here, $K_{f_{j}u_{j}}\in R^{N\times M}$ denotes the cross-covariance computed by evaluating $k_{j}\left(\cdot ,\cdot \right)$ between input samples and inducing points. Similarly, $K_{u_{j}u_{j}}\in R^{M\times M}$ represents the covariance matrix between inducing points. By conditioning on the inducing variables $u_{j}$, the marginal distribution over the latent function values $f_{j}$ is given by:

(3) $$p\left(f_{j}|u_{j}\right)=N\left(f_{j}|K_{f_{j}u_{j}}K_{u_{j}u_{j}}^{-1}u_{j},K_{f_{j}f_{j}}-K_{f_{j}u_{j}}K_{u_{j}u_{j}}^{-1}K_{u_{j}f_{j}}\right),$$

and the prior on $u_{j}$ is:

(4) $$p\left(u_{j}\right)=N\left(u_{j}|0,K_{u_{j}u_{j}}\right).$$

In general, the posterior distribution $p(\hat{f},\hat{u}|y)$ is intractable due to the non-conjugacy of the likelihood with respect to the priors ($\hat{u}={\left[u_{1}^{⊤},\ldots ,u_{J}^{⊤}\right]}^{⊤}\in R^{MJ}$). Then, we approximate the posterior using variational inference with a parameterized distribution $p\left(\hat{f},\hat{u}|y\right)\approx \tilde{q}\left(\hat{f},\hat{u}\right)$ [23]:

(5) $$\tilde{q}\left(\hat{f},\hat{u}\right)=p\left(\hat{f}|\hat{u}\right)\tilde{q}\left(\hat{u}\right)=\prod_{j=1}^{J}p\left(f_{j}|u_{j}\right)\tilde{q}\left(u_{j}\right),$$

where $\tilde{q}\left(u_{j}\right)$ is a variational Gaussian distribution over the inducing variables and:

(6) $$\tilde{q}\left(\hat{u}\right)=\prod_{j=1}^{J}\tilde{q}\left(u_{j}\right)=\prod_{j=1}^{J}N\left(u_{j}|{\hat{\mu }}_{j},{\hat{V}}_{j}\right),$$

with ${\hat{\mu }}_{j}\in R^{M}$ and ${\hat{V}}_{j}\in R^{M\times M}$. These parameters are optimized by maximizing an Evidence Lower Bound (ELBO), which provides a tractable surrogate to the marginal likelihood. Assuming that the data points $x_{n}$ are independently and identically distributed, such an ELBO can be formulated as [47]:

(7) $$L=\sum_{n=1}^{N}E_{\tilde{q}\left(f_{1}\right),\ldots ,\tilde{q}\left(f_{J}\right)}\left[\log p(y_{n}|\theta_{1}\left(x_{n}\right),\ldots ,\theta_{J}\left(x_{n}\right))\right]-\sum_{j=1}^{J}D_{\mathrm{KL}}\left(\tilde{q}\left(u_{j}\right)\;∥\;p\left(u_{j}\right)\right),$$

where $D_{\mathrm{KL}}\left(\cdot ∥\cdot \right)$ is the Kullback–Leibler divergence and $\tilde{q}\left(f_{j}\right)=\int p\left(f_{j}|u_{j}\right)\tilde{q}\left(u_{j}\right)\;du_{j}$.

Still, the standard CGP assumes independent GP priors for each likelihood parameter. The latter assumption is often unrealistic in multi-annotator scenarios, where annotators may share common biases or information sources [31]. To capture these dependencies, we employ a shared latent-factor structure [48]:

(8) $${\overset{˘}{f}}_{j}\left(x_{n}\right)=\sum_{q=1}^{Q}w_{j,q}\vartheta_{q}\left(x_{n}\right),$$

where ${\overset{˘}{f}}_{j}:R^{P}\to R$, $\vartheta_{q}\left(\cdot \right)∼\mathrm{GP}\left(0,\kappa_{q}\left(\cdot ,\cdot \right)\right)$, and $w_{j,q}\in R$. Let $\overset{˘}{\theta }={\left[\theta_{1}^{⊤},\ldots ,\theta_{J}^{⊤}\right]}^{⊤}\in R^{NJ}$ be the sample-dependent model parameter vector, $\overset{˘}{f}={\left[{\overset{˘}{f}}_{1},\ldots ,{\overset{˘}{f}}_{J}\right]}^{⊤}\in R^{NJ}$, ${\overset{˘}{f}}_{j}={\left[{\overset{˘}{f}}_{j}\left(x_{1}\right),\ldots ,{\overset{˘}{f}}_{j}\left(x_{N}\right)\right]}^{⊤}\in R^{N}$, and q∈{1,2,…,Q}. Therefore, the Correlated Chained Gaussian Process (CCGP) framework naturally emerges when the joint distribution in Equation (1) is reformulated to explicitly model dependencies across outputs through a chained, conditionally correlated structure:

(9) $$p\left(y,\overset{˘}{f},\overset{˘}{u}|X\right)=p\left(y|\overset{˘}{\theta }\right)\prod_{j=1}^{J}p\left({\overset{˘}{f}}_{j}|\overset{˘}{u}\right)p\left(\overset{˘}{u}\right).$$

For each $\vartheta_{q}\left(\cdot \right)$, we introduce pseudo-variables ${\overset{˘}{u}}_{q}={\left[\vartheta_{q}\left({\overset{˘}{z}}_{1}^{q}\right),\ldots ,\vartheta_{q}\left({\overset{˘}{z}}_{M}^{q}\right)\right]}^{⊤}\in R^{M}$, by evaluating $\vartheta_{q}\left(\cdot \right)$ at ${\overset{˘}{Z}}_{q}=\left[{\overset{˘}{z}}_{1}^{q},\ldots ,{\overset{˘}{z}}_{M}^{q}\right]\in R^{M\times P}$. Likewise, $\overset{˘}{u}={\left[{\overset{˘}{u}}_{1}^{⊤},\ldots ,{\overset{˘}{u}}_{Q}^{⊤}\right]}^{⊤}\in R^{QM}$, then:

(10) $$p\left({\overset{˘}{f}}_{j}|\overset{˘}{u}\right)=N\left({\overset{˘}{f}}_{j}\;|\;K_{{\overset{˘}{f}}_{j}\overset{˘}{u}}K_{\overset{˘}{u}\overset{˘}{u}}^{-1}\overset{˘}{u},\;K_{{\overset{˘}{f}}_{j}{\overset{˘}{f}}_{j}}-K_{{\overset{˘}{f}}_{j}\overset{˘}{u}}K_{\overset{˘}{u}\overset{˘}{u}}^{-1}K_{\overset{˘}{u}{\overset{˘}{f}}_{j}}\right),$$

(11) $$p\left(\overset{˘}{u}\right)=N\left(\overset{˘}{u}|0,K_{\overset{˘}{u}\overset{˘}{u}}\right)=\prod_{q=1}^{Q}N\left({\overset{˘}{u}}_{q}|0,K_{{\overset{˘}{u}}_{q}{\overset{˘}{u}}_{q}}\right);$$

where $K_{\overset{˘}{u}\overset{˘}{u}}\in R^{QM\times QM}$ is block-diagonal, with $K_{{\overset{˘}{u}}_{q}{\overset{˘}{u}}_{q}}\in R^{M\times M}$ computed from $\kappa_{q}\left(\cdot ,\cdot \right)$. The covariance matrix $K_{{\overset{˘}{f}}_{j}{\overset{˘}{f}}_{j}}\in R^{N\times N}$ has elements $\sum_{q=1}^{Q}w_{j,q}^{2}\kappa_{q}\left(x_{n},x_{n^{\prime }}\right)$.

Similarly, $K_{{\overset{˘}{f}}_{j}\overset{˘}{u}}=\left[K_{{\overset{˘}{f}}_{j}{\overset{˘}{u}}_{1}},\ldots ,K_{{\overset{˘}{f}}_{j}{\overset{˘}{u}}_{Q}}\right]\in R^{N\times QM}$, where $K_{{\overset{˘}{f}}_{j}{\overset{˘}{u}}_{q}}\in R^{N\times M}$ holds elements $w_{j,q}\kappa_{q}\left(x_{n},{\overset{˘}{z}}_{m}^{q}\right)$, with m∈{1,2,…,M}. Similar to the CGP, the posterior distribution of the CCGP, denoted as $p(\overset{˘}{f},\overset{˘}{u}|y)$, is generally intractable in closed form. Consequently, it is approximated using a parameterized variational distribution, i.e., $p\left(\overset{˘}{f},\overset{˘}{u}|y\right)\approx \tilde{q}\left(\overset{˘}{f},\overset{˘}{u}\right)$, following the approach in [23]:

(12) $$\tilde{q}\left(\overset{˘}{f},\overset{˘}{u}\right)=p\left(\overset{˘}{f}|\overset{˘}{u}\right)\;\tilde{q}\left(\overset{˘}{u}\right)=\prod_{j=1}^{J}p\left({\overset{˘}{f}}_{j}|\overset{˘}{u}\right)\prod_{q=1}^{Q}\tilde{q}\left({\overset{˘}{u}}_{q}\right),$$

where $\tilde{q}\left({\overset{˘}{u}}_{q}\right)=N\left({\overset{˘}{u}}_{q}|{\overset{˘}{\mu }}_{q},{\overset{˘}{V}}_{q}\right)$ and $\tilde{q}\left(\overset{˘}{u}\right)=N\left(\overset{˘}{u}|\overset{˘}{\mu },\overset{˘}{V}\right)$. In addition, $\overset{˘}{\mu }=\left[{\overset{˘}{\mu }}_{1}^{⊤},\ldots ,{\overset{˘}{\mu }}_{Q}^{⊤}\right]\;\in \;R^{QM}$ (${\overset{˘}{\mu }}_{q}\in R^{M}$) and $\overset{˘}{V}\in R^{QM\times QM}$ is a block-diagonal matrix holding covariance blocks ${\overset{˘}{V}}_{q}\;\in \;R^{M\times M}.$ We then approximate the joint posterior over all latent functions and their corresponding inducing variables using a factorized variational distribution. This approximation leads to the following ELBO:

(13) $$L=\sum_{n=1}^{N}E_{\tilde{q}\left({\overset{˘}{f}}_{1}\right),\ldots ,\tilde{q}\left({\overset{˘}{f}}_{J}\right)}\left[\log p(y_{n}|\theta_{1}\left(x_{n}\right),\ldots ,\theta_{J}\left(x_{n}\right))\right]\;-\;\sum_{q=1}^{Q}D_{\mathrm{KL}}\left(\tilde{q}\left({\overset{˘}{u}}_{q}\right)\;∥\;p\left({\overset{˘}{u}}_{q}\right)\right),$$

where:

(14) $$\tilde{q}\left({\overset{˘}{f}}_{j}\right)=N\left({\overset{˘}{f}}_{j}|K_{{\overset{˘}{f}}_{j}\overset{˘}{u}}K_{\overset{˘}{u}\overset{˘}{u}}^{-1}\overset{˘}{\mu },\;K_{{\overset{˘}{f}}_{j}{\overset{˘}{f}}_{j}}+K_{{\overset{˘}{f}}_{j}\overset{˘}{u}}K_{\overset{˘}{u}\overset{˘}{u}}^{-1}\left(\overset{˘}{V}-K_{\overset{˘}{u}\overset{˘}{u}}\right)K_{\overset{˘}{u}\overset{˘}{u}}^{-1}K_{\overset{˘}{u}{\overset{˘}{f}}_{j}}\right).$$

The first term in Equation (13) encourages the variational posterior to explain the observed labels, while the second penalizes deviation from the GP prior over latent factors.

### 2.3. CCGP-Based Crowd Learning and Localized Annotator Trustworthiness

Consider a multi-annotator dataset $D_{R}=\left\{X\in R^{N\times P},Y\in R^{N\times R}\right\}$, where X denotes the input features and Y is the corresponding label matrix provided by *R* annotators with unknown and potentially heterogeneous levels of expertise. The entry $y_{n}^{r}\in Y$ represents the label assigned by annotator *r* to the *n*-th sample. Since annotators may not label all samples, the matrix Y can contain missing values. Let R⊂{1,…,N}×{1,…,R} denote the set of observed annotation indices. Then, for each (n,r)∈R, a label $y_{n}^{r}$ is observed, while for (n,r)∉R, the annotation is considered missing and excluded.

Here, we propose a CCGP-based crowd learning framework for multi-annotator regression tasks, referred to as MAR-CCGP. This approach is designed with two primary objectives: (i) to model each annotator’s performance as a localized function of the input space, thereby capturing annotator-specific trustworthiness across different regions; and (ii) to accurately infer the true label $y_{*}\in Y$ for a new input $x_{*}\in R^{P}$. Notably, the method operates in a fully unsupervised manner with respect to annotator reliability, no additional supervision regarding annotator expertise, experience, or consistency is assumed.

Conversely, for real-value outputs, we follow the multi-annotator approach in [49], where each $y_{n}^{r}$ is considered as a corrupted version of the estimated hidden ground truth ${\hat{y}}_{n}\in R$, yielding:

(15) $$p\left(Y|\overset{˘}{\theta }\right)=\prod_{(n,r)\in R}N\left(y_{n}^{r}|{\hat{y}}_{n},{\overset{˘}{v}}_{n}^{r}\right)$$

where ${\overset{˘}{v}}_{n}^{r}\in R^{+}$ denotes the error variance associated with the *r*-th annotator for instance *n*. In our MAR-CCGP framework, each parameter of the likelihood in $\overset{˘}{\theta }$ is linked to an LF ${\overset{˘}{f}}_{j}\left(\cdot \right)$, as defined in Equation (8). Specifically, our model employs J=R+1 LFs: one dedicated to capturing the latent ground truth, and the remaining *R* to characterize the input-dependent error variances of each annotator:

(16) $$\begin{array}{cc} {\hat{y}}_{n}={\overset{˘}{f}}_{1}\left(x_{n}\right)= & \sum_{q=1}^{Q}\omega_{1,q}\vartheta_{q}\left(x_{n}\right), \end{array}$$

(17) $$\begin{array}{cc} {\overset{˘}{v}}_{n}^{r}=\exp\left(f_{l_{r}}\left(x_{n}\right)\right)= & \exp\left(\sum_{q=1}^{Q}\omega_{l_{r},q}\vartheta_{q}\left(x_{n}\right)\right); \end{array}$$

where $\forall l_{r}\in \left\{2,\ldots ,J\right\}$. Note that an exponential transformation is applied to the corresponding LF in Equation (17) to ensure that the annotator-specific variance remains strictly positive, i.e., ${\overset{˘}{v}}_{n}^{r}>0$.

Afterward, an ELBO-based optmization from Equation (13) is introduced for our MAR-CCGP, as follows:

(18) $$L=\sum_{(n,r)\in R}E_{\tilde{q}\left({\overset{˘}{f}}_{1}\right),\ldots ,\tilde{q}\left({\overset{˘}{f}}_{l_{R}}\right)}\left[\log\left(N(y_{n}^{r}|{\hat{y}}_{n},{\overset{˘}{v}}_{n}^{r})\right)\right]\;-\;\sum_{q=1}^{Q}D_{\mathrm{KL}}\left(\tilde{q}\left({\overset{˘}{u}}_{q}\right)\;∥\;p\left({\overset{˘}{u}}_{q}\right)\right),$$

where $\tilde{q}\left({\overset{˘}{f}}_{j}\right)$ for $j\in \{1,l_{1},l_{2},\ldots ,l_{R}\}$ denotes the variational marginal over the latent function values, as defined in Equation (14); $\tilde{q}\left({\overset{˘}{u}}_{q}\right)$ represents the variational distribution over the inducing variables (see Equation (12)); and $p({\overset{˘}{u}}_{q})$ corresponds to the GP prior over the inducing variables, given in Equation (11).

In turn, given a new input sample $x_{*}$, our goal is to compute the predictive mean and variance for both the estimated ground truth ${\hat{y}}_{*}$ and the corresponding annotator-specific error variances ${\overset{˘}{v}}_{*}^{r}$. Specifically, as we defined the ground truth prediction as ${\hat{y}}_{*}={\overset{˘}{f}}_{1}\left(x\right)$, the posterior distribution over ${\overset{˘}{f}}_{1}\left(x\right)$ is given by:

(19) $$\tilde{q}\left({\overset{˘}{f}}_{1}\left(x_{*}\right)\right)=\int p\left({\overset{˘}{f}}_{1}\left(x_{*}\right)|{\overset{˘}{u}}_{q}\right)\tilde{q}\left({\overset{˘}{u}}_{q}\right)d{\overset{˘}{u}}_{q}=N\left({\overset{˘}{f}}_{1}\left(x_{*}\right)|{\overset{˘}{\mu }}_{1,*},\sigma_{1,*}^{2}\right);$$

where:

(20) $$\begin{array}{cc} E[{\hat{y}}_{*}]= & {\overset{˘}{\mu }}_{1,*}\in R, \end{array}$$

(21) $$\begin{array}{cc} \mathrm{var}[{\hat{y}}_{*}]= & \sigma_{*}^{2}\in R^{+}. \end{array}$$

Similarly, due to the exponential transformation in Equation (17), the posterior distribution of the annotator-specific variance ${\overset{˘}{v}}_{*}^{r}$ follows a log-normal distribution. Its parameters are determined by the predictive mean ${\overset{˘}{\mu }}_{l_{r},*}$ and variance $\sigma_{l_{r},*}^{2}$ in $\tilde{q}\left({\overset{˘}{f}}_{l_{r}}\left(x_{*}\right)\right)$, yielding:

(22) $$\begin{array}{cc} E[{\overset{˘}{v}}_{*}^{r}]= & \exp\left({\overset{˘}{\mu }}_{l_{r},*}+\frac{\sigma_{l_{r},*}^{2}}{2}\right)\in R^{+}, \end{array}$$

(23) $$\begin{array}{cc} \mathrm{var}[{\overset{˘}{v}}_{*}^{r}]= & \exp\left(2{\overset{˘}{\mu }}_{l_{r},*}+\sigma_{l_{r},*}^{2}\right)\left(\exp\left(\sigma_{l_{r},*}^{2}\right)-1\right)\in R^{+}. \end{array}$$

Finally, the proposed MAR-CCGP framework enables the assessment of localized annotator trustworthiness through a probabilistic reliability score derived from the model’s posterior predictions. For each annotator *r* and sample $x_{n}$, the trustworthiness score $T_{r}\left(x_{n}\right)$ is defined as:

(24) $$T_{r}\left(x_{n}\right)=\exp\left(-\frac{|y_{n}^{r}-{\hat{y}}_{n}{|}_{2}^{2}}{2{\left({\overset{˘}{v}}_{n}^{r}\right)}^{\gamma }}\right),$$

where ${\hat{y}}_{n}$ denotes the predicted ground truth for input sample $x_{n}$, and ${\overset{˘}{v}}_{n}^{r}$ is the estimated annotator-specific variance as in Equations (20) and (22), respectively. The exponent γ∈(0,1] modulates the sensitivity to the model’s predicted uncertainty, acting as a sublinear scaling factor. Setting γ=0.5 was found to stabilize the trustworthiness score in regions of low predicted variance, preventing numerical explosions and yielding better alignment with empirical annotator behavior. This formulation corresponds to the scaled likelihood of the observed annotation $y_{n}^{r}$ under the model’s uncertainty, offering a principled, data-dependent trust metric for each annotator at a given input location.

## 3. Experimental Set-Up

This section details the datasets, preprocessing pipeline, model-training configuration, baseline methods, and evaluation metrics used in all experiments. To evaluate the effectiveness of the proposed MAR-CCGP framework in modeling information from multiple annotators, we conduct a series of experiments using both semi-synthetic and real-world datasets. The semi-synthetic benchmarks provide a controlled environment with access to ground-truth labels, enabling rigorous assessment of the model’s ability to infer annotator trustworthiness. In contrast, the real-world dataset—comprising sensory evaluations of cacao products (see the LUKER-CACAO dataset description in Section 2.1)—lacks ground-truth annotations, making it an ideal scenario to assess the framework’s capabilities in consensus estimation and uncertainty quantification.

### 3.1. Semi-Synthetic Dataset Annotation Simulation

We evaluate our MAR-CCGP approach under controlled conditions employing regression datasets from the University of California Irvine (UCI) machine learning repository (see https://archive.ics.uci.edu/datasets, accessed on 3 July 2025 and Table 4). Each dataset provides continuous targets and input features, enabling the generation of simulated multi-annotator labels with structured noise profiles.

Moreover, to simulate annotator-specific variability, we adopt the following scheme:

– The input data are standardized and then projected into a two-dimensional space using Uniform Manifold Approximation and Projection (UMAP) [50] to preserve local data structure by minimizing the cross-entropy between high-dimensional and low-dimensional fuzzy simplicial representations.
– A *K*-means algorithm with *C* clusters is then applied to the UMAP projection to derive pseudo-contextual input space partitions [30] as latent indicators of instance-specific difficulty or domain shifts, used to modulate annotator behavior.
– Let $c_{n}\in \left\{1,\ldots ,C\right\}$ denote the cluster assignment associated with the input $x_{n}$, and let $y_{n}$ represent the corresponding ground-truth regression value. The simulated label assigned by annotator *r* to instance *n* is then generated as follows:
  (25) $$y_{n}^{r}=y_{n}+\epsilon_{n}^{r},\;\epsilon_{n}^{r}∼N\left(\epsilon_{n}^{r}|0,{\tilde{\sigma }}_{r,c_{n}}^{2}\right);$$
  where ${\tilde{\sigma }}_{r,c_{n}}^{2}\in R^{+}$ is the annotator-specific error variance for cluster $c_{n}$. To ensure interpretability and reproducibility, each annotator’s variance profile across clusters is defined in terms of a fixed Signal-to-Noise Ratio (SNR) in dB. Then, the multiple annotator target matrix for regression tasks $Y\in R^{N\times R}$ is built as in Equation (25).

**Figure 4:**
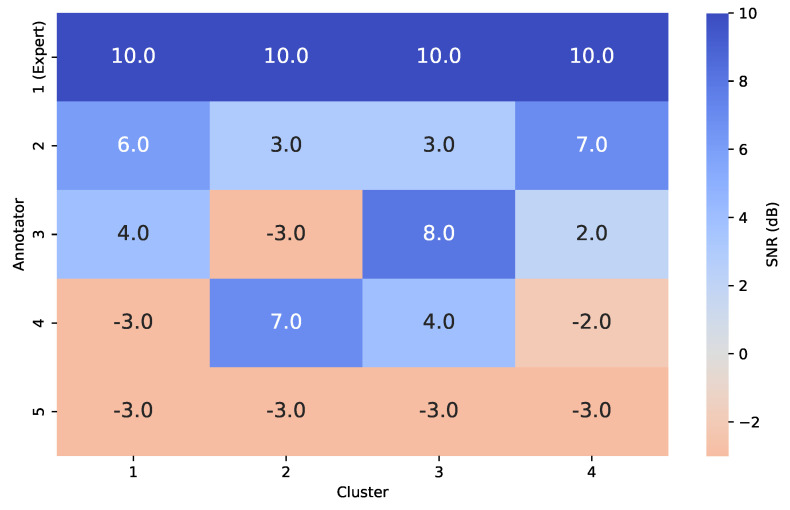
Signal−to−Noise Ratio (SNR, in dB) matrix used in the semi-synthetic annotation simulation to model annotator-specific variance across clusters. Annotator 1 serves as a uniformly reliable expert, while the remaining annotators exhibit cluster-dependent variability in labeling accuracy.

By varying the SNR across annotators and data clusters, we construct distinct variance profiles wherein certain annotators exhibit high reliability in specific regions of the input space and low reliability in others. This setup is intended to emulate real-world, non-stationary labeling behavior. For concreteness in our semi-synthetic data experiments, we fix the number of clusters to C=4 and the number of annotators to R=5. The SNR values are varied according to the profiles shown in Figure 4. One annotator is designated as an expert, consistently exhibiting an SNR of 10dB across all data clusters.

### 3.2. Quality Assessment, Method Comparison, and Training Details

To evaluate whether the model-derived reliability scores accurately reflect annotator behavior, we aggregate both the predicted trustworthiness scores and empirical performance metrics across annotators and clusters. Specifically, for each annotator, we compute the average trustworthiness score within each cluster (see Equation (24)), defined as:

(26) $${\bar{T}}_{r}\left(\Omega_{\tilde{c}}\right)=\frac{1}{|\Omega_{\tilde{c}}|}\sum_{x_{n}\in \Omega_{\tilde{c}}}T_{r}\left(x_{n}\right),$$

where $\Omega_{\tilde{c}}$ denotes the set of input instances in cluster $\tilde{c}\in C$. For comparison, we compute the empirical coefficient of determination between annotator labels and model-predicted ground truth in the same regions:

(27) $$R_{r}^{2}\left(\Omega_{\tilde{c}}\right)=1-\frac{\sum_{\forall n:x_{n}\in \Omega_{\tilde{c}}}{∥y_{n}^{r}-y_{n}∥}_{2}^{2}}{\sum_{\forall n:x_{n}\in \Omega_{\tilde{c}}}{∥y_{n}^{r}-\bar{y}∥}_{2}^{2}},$$

where $\bar{y}=\frac{1}{|\Omega_{\tilde{c}}|}\sum_{n}y_{n},$∀$n:x_{n}\in \Omega_{\tilde{c}}.$

We benchmarked the proposed MAR-CCGP against three representative baselines, each embodying a distinct strategy for handling multi-annotator learning. As outlined in Table 5, the GPR-GT model serves as an optimistic upper bound, leveraging a GP regressor [30] trained directly on ground truth targets. This approach bypasses the inherent complexities of annotator-induced variability, thereby providing a best-case performance scenario. In contrast, the GPR-AVG represents a naive lower-bound reference, where annotations are averaged per instance to form pseudo-targets for GP training [23]. This method assumes uniform annotator reliability and fails to account for noise or systematic biases. A more nuanced alternative is offered by LKAAR, which simultaneously models annotator-specific noise and bias while learning an input-dependent reliability function via kernel alignment [31]. Notably, both LKAAR and our MAR-CCGP are among the few methods capable of modeling annotator consistency as a function of the input space.

Also, to quantitatively assess the predictive performance of the MAR-CCGP model for the semi-synthetic datasets, we include three standard regression measures: Mean Squared Error (MSE), Mean Absolute Error (MAE), and Mean Absolute Percentage Error (MAPE):

(28) $$\begin{array}{cc} \mathrm{MSE}(y,\hat{y}) & =\frac{1}{N}\sum_{n=1}^{N_{\mathrm{test}}}{\left(y_{n}-{\hat{y}}_{n}\right)}^{2}, \end{array}$$

(29) $$\begin{array}{cc} \mathrm{MAE}(y,\hat{y}) & =\frac{1}{N}\sum_{n=1}^{N_{\mathrm{test}}}\left|y_{n}-{\hat{y}}_{n}\right|, \end{array}$$

(30) $$\begin{array}{cc} \mathrm{MAPE}(y,\hat{y}) & =\frac{100}{N}\sum_{n=1}^{N_{\mathrm{test}}}\left|\frac{y_{n}-{\hat{y}}_{n}}{y_{n}+\overset{˘}{\epsilon }}\right|, \end{array}$$

(31) $$\begin{array}{cc} R^{2}\left(y,\hat{y}\right) & =1-\frac{\sum_{n=1}^{N}{\left(y_{n}-{\hat{y}}_{n}\right)}^{2}}{\sum_{n=1}^{N_{\mathrm{test}}}{\left(y_{n}-\bar{y}\right)}^{2}}, \end{array}$$

where $y_{n}\in y$ is the true target (available in semi-synthetic scenarios) and ${\hat{y}}_{n}\in \hat{y}$ is the predicted ground truth. $\overset{˘}{\epsilon }>0$ is a small constant added to avoid division by zero when $y_{n}\approx 0$ (set to $\overset{˘}{\epsilon }=10^{-6}$ in our experiments), and $\bar{y}=\frac{1}{N}\sum_{n=1}^{N}y_{n}$ denotes the sample mean of the ground truth values.

All experiments were conducted on the Kaggle platform (*Kaggle, Inc.*, San Francisco, CA, USA) using GPU-enabled notebooks. Each session provided access to a single NVIDIA Tesla P100 GPU (16GB VRAM; *NVIDIA Corporation*, Santa Clara, CA, USA), 30 GB of RAM, and 4 vCPUs from an Intel Xeon CPU @ 2.20GHz (*Intel Corporation*, Santa Clara, CA, USA). The code-base was developed in Python 3.11.11 (*Python Software Foundation*, Wilmington, DE, USA), and all models were executed in the default Kaggle environment, with package versions listed in the notebook dependencies. Our MAR-CCGP model was implemented using GPflow 2.10.0 atop TensorFlow 2.18.0 (*Google LLC*, Mountain View, CA, USA). Dimensionality reduction and clustering were handled by cuML 25.2.1 (*NVIDIA Corporation*, Santa Clara, CA, USA), while preprocessing relied on scikit-learn 1.2.1 (*scikit-learn developers*, open-source community, global). Additional tools included NumPy 1.26.4 (*Travis Oliphant et al.*, open-source community, USA) Pandas 2.2.2 (*Wes McKinney et al.*, open-source community, USA), and Matplotlib 3.8.4 (*John D. Hunter et al.*, open-source community, USA) for analysis and visualization.

All GP-based approaches and the regression components within LKAAR used sparse variational inference with M=200 inducing points and a mini-batch size of 128. Inducing inputs were initialized via *k*-means clustering. A squared exponential kernel was used in all cases. Training was carried out using the Adam optimizer with a learning rate of $10^{-2}$ for up to 1000 steps. If the ELBO did not improve for 20 consecutive iterations, the learning rate was halved to a minimum of $10^{-6}$. Early stopping was triggered if no improvement was observed over 500 iterations.

**Figure 5:**
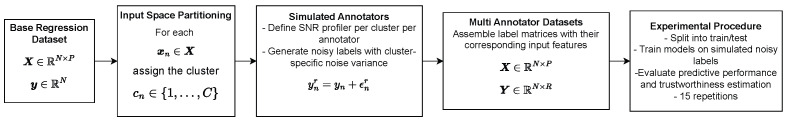
Semi-synthetic datasets MAR-CCGP experimental set-up.

**Figure 6:**
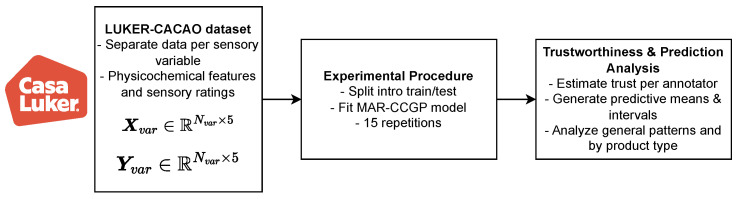
LUKER-CACAO dataset MAR-CCGP experimental set-up.

Each experiment was repeated over 15 randomized train/test splits (70/30). For the semi-synthetic datasets, splits were stratified by the UMAP-based cluster labels to preserve input-space diversity across folds. In the cacao-based real-world dataset (LUKER-CACAO), where no cluster labels were available, standard random splits were applied. Random seeds were fixed for all runs to ensure reproducibility. Code and full experiment notebooks for semi-synthetics datasets are publicly available at: https://github.com/UN-GCPDS/python-gcpds.luker_multiple_annotators, accessed on 3 July 2025. Access to the LUKER-CACAO dataset is restricted owing to copyright limitations established by the Casa Luker organization. The experimental set-ups for the semi-synthetic datasets and the LUKER-CACAO dataset are illustrated in Figure 5 and Figure 6, respectively.

## 4. Results and Discussion

### 4.1. Semi-Synthetic Datasets Results

We first evaluate all models using the semisynthetic benchmark datasets described in Table 4. These datasets allow controlled experimentation with known ground truth, enabling a detailed assessment of both predictive accuracy and the quality of learned annotator reliability.

**Figure 7:**
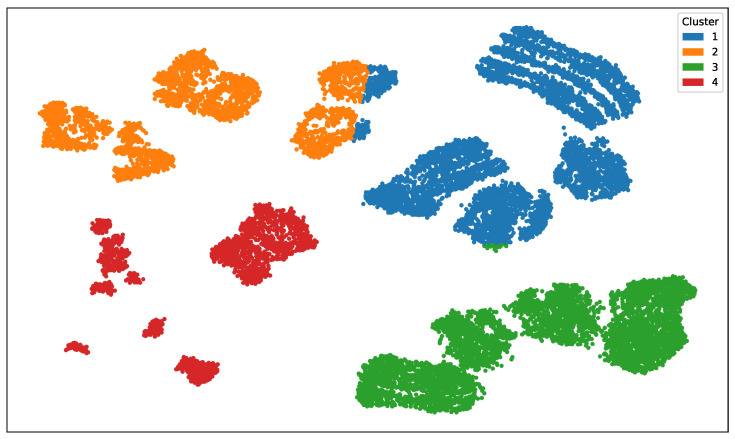
UMAP compresses any high-dimensional dataset into two latent coordinates that keep close neighbours together. Here, we applied it to the Bike Sharing data as an illustrative case: each point is an hourly record, plotted by its two UMAP components. Running *k*-means with k!=!4 on the embedded points produces the four color-coded clusters you see. Because UMAP preserves neighborhood structure, each cluster represents a coherent subset of samples that share similar covariate profiles (e.g., comparable weather or calendar conditions in this dataset, but more generally any common context in other data). In our simulation framework, we exploit this idea of “context”: every cluster is assigned its own annotator-noise model, so label accuracy can vary according to which region of the data manifold a sample belongs to.

**Figure 8:**
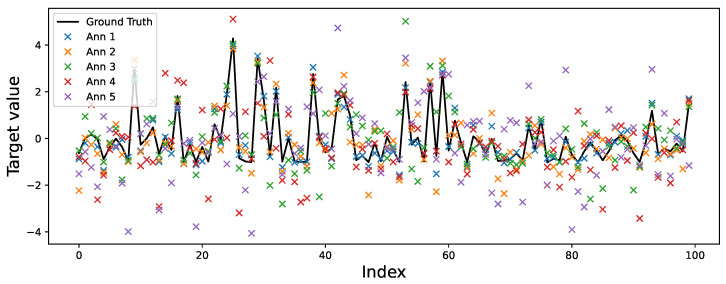
Simulated multi-annotator labels for a subset of the Bike Sharing dataset. The solid black line represents the true target values, while each color-coded scatter indicates the noisy annotations from five simulated annotators. Samples are sorted by cluster to highlight regional reliability patterns: consistent deviations from the ground truth reveal how annotator accuracy varies across input regions, as defined by the SNR matrix.

To simulate non-stationary annotator behavior, we followed the procedure outlined in Section 3.1. Ground truth labels were corrupted using structured noise profiles defined by a Signal-to-Noise Ratio (SNR) matrix, which assigns fixed reliability levels per annotator and input cluster. As shown in Figure 4, Annotator 1 is modeled as an expert with uniformly high reliability (10 dB across all clusters). In contrast, Annotators 2–5 display varying degrees of cluster-dependent behavior. For example, Annotator 2 is more reliable in Clusters 1 and 4 (6 and 7 dB), while Annotator 3 peaks in Cluster 3 (8 dB) but is unreliable in Cluster 2 (−3 dB). Annotator 4 demonstrates high reliability in Cluster 2 (7 dB) and moderate accuracy in Cluster 3 (4 dB), yet performs poorly in Clusters 1 and 4. Annotator 5 acts as a noisy rater with consistently low SNR (−3 dB across all regions), representing a challenging labeling scenario. Figure 7 illustrates the UMAP-based clustering for the Bike Sharing dataset, and Figure 8 displays the resulting noisy annotations alongside the ground truth.

All models were evaluated over 15 randomized 70/30 train/test splits, stratified by the clusters used in the annotator simulation. We report the mean and standard deviation of test-set metrics: MSE, MAE, MAPE, $R^{2}$. Results are presented in Table 6.

Across most datasets, MAR-CCGP achieves the strongest overall performance across all regression metrics—MSE, MAE, MAPE, and $R^{2}$—demonstrating its capacity to model input-dependent annotator reliability and structured inter-annotator noise. It obtains the lowest errors and highest $R^{2}$ in four out of five benchmarks, with notable advantages in challenging datasets like Concrete Strength and Auto MPG. While GPR-GT, trained with access to clean ground truth, retains a slight edge in the Yacht Hydrodynamics task, MAR-CCGP closely matches its accuracy despite relying solely on noisy annotations. An exception is found in the Bike Sharing dataset, where LKAAR achieves the best MAPE score, and MAR-CCGP lags slightly behind in that metric—highlighting a sensitivity to error scaling in high-variance settings. GPR-AVG, which naïvely averages annotations, consistently underperforms due to its inability to model contextual noise patterns. Meanwhile, LKAAR, although capable of estimating local reliability, lacks mechanisms to model annotator correlations or structured uncertainty, leading to lower accuracy and higher variance in most tasks. These results underscore the robustness of MAR-CCGP’s joint modeling approach and its suitability for learning under sparse, noisy, and context-dependent supervision.

**Figure 9:**
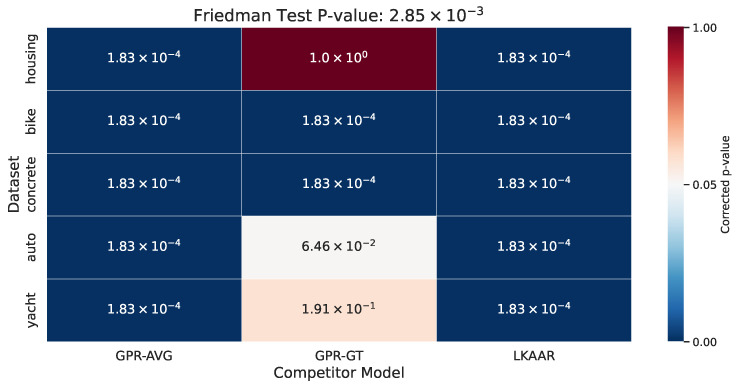
Corrected *p*-values from Wilcoxon signed-rank tests comparing MAR-CCGP to each competitor model using $R^{2}$ scores across all semi-synthetic datasets. Bonferroni correction was applied (3 comparisons per dataset). The Friedman test reported a global *p*-value of $2.85\times 10^{-3}$, confirming significant performance differences.

To further validate the observed performance differences, we conducted a statistical significance analysis on the $R^{2}$ metric, chosen for its interpretability and robustness across varying label scales. A Friedman test across all datasets yielded a *p*-value of $2.85\times 10^{-3}$, indicating significant differences among the models. Subsequently, we performed pairwise Wilcoxon signed-rank tests between MAR-CCGP and each competing method, applying Bonferroni correction (three comparisons per dataset). The results, summarized in Figure 9, show that MAR-CCGP significantly outperforms both GPR-AVG and LKAAR across all benchmarks. While the comparison with GPR-GT yields non-significant differences in some datasets (e.g., p=0.06464 for Auto MPG, p=0.19116 for Yacht), this is expected, as GPR-GT serves as an idealized upper bound leveraging clean ground truth unavailable to our model. These findings reinforce MAR-CCGP’s strong empirical performance and competitive advantage under realistic noisy supervision.

To evaluate MAR-CCGP’s ability to recover localized annotator trustworthiness, we applied the assessment framework described in Section 2.3. Specifically, for each dataset, we partitioned the input space into clusters and computed two cluster-level matrices: (1) the empirical coefficient of determination $R_{r}^{2}\left(\Omega_{\tilde{c}}\right)$, which quantifies the agreement between each annotator’s labels and the inferred ground truth, and (2) the average localized trustworthiness score ${\bar{T}}_{r}\left(\Omega \tilde{c}\right)$ derived from the model’s posterior predictive distribution. These metrics enable a direct comparison between empirical annotator behavior and the model’s estimated reliability across different regions of the input space.

**Figure 10:**
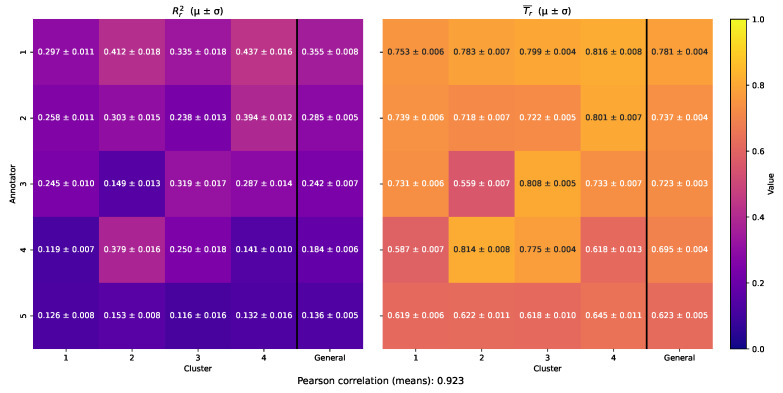
Cluster-specific evaluation of annotator reliability in the Bike Sharing simulation. The dataset is first partitioned into four regimes (e.g., cold/low-usage vs. warm/high-usage) via UMAP + *k*-means. (**Left**) Empirical coefficient of determination $R_{r}^{2}\left(\Omega_{\tilde{c}}\right)$ between each annotator’s noisy labels and the true (uncorrupted) target, computed within each cluster. (**Right**) MAR-CCGP–inferred trustworthiness scores ${\bar{T}}_{r}\left(\Omega_{\tilde{c}}\right)$ for each annotator and cluster. Annotator 1’s uniformly high values illustrate global expertise, while the other annotators’ scores fluctuate according to cluster-dependent noise levels introduced in our simulation.

Figure 10 presents the cluster-wise empirical $R_{r}^{2}\left(\Omega_{\tilde{c}}\right)$ scores (left) alongside the MAR-CCGP trustworthiness estimates ${\bar{T}}_{r}\left(\Omega_{\tilde{c}}\right)$ (right) for the *Bike Sharing* dataset. As expected from its uniform SNR profile, Annotator 1 is the most accurate across all clusters, reaching the highest $R^{2}$ values (e.g., 0.816±0.008 in Cluster 4), and the model correctly assigns the highest trust scores to this annotator (e.g., 0.437±0.016 in Cluster 4). Annotators 2–5 exhibit varying degrees of cluster-specific reliability, with MAR-CCGP successfully recovering these patterns. For instance, Annotator 4, the most competent in Cluster 2 ($R^{2}=0.814\pm 0.008$), is assigned the second-highest trust score in that region (0.379±0.016), despite being less accurate elsewhere (e.g., $R^{2}=0.587\pm 0.007$ in Cluster 1). These results demonstrate the model’s ability to localize annotator reliability even under heterogeneous noise structures.

**Figure 11:**
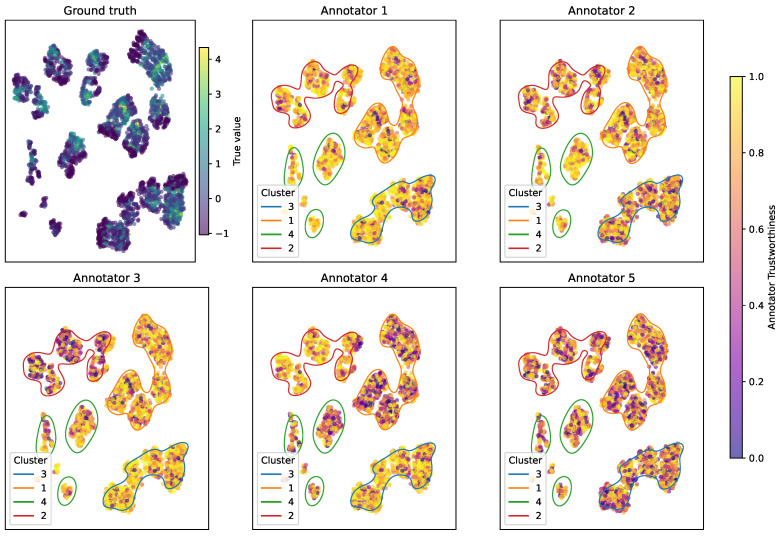
Per-sample reliability maps for each annotator in the Bike Sharing case. (**Top–left**) The true (uncorrupted) target values. (**Panels 2–6**) MAR-CCGP’s predicted trustworthiness for Annotators 1–5 on each sample (shading from low to high). Annotator 1 remains consistently reliable across the entire input space, whereas the others exhibit localized drops in trust corresponding to the simulated SNR regimes.

To further validate this alignment, we computed the squared Pearson correlation between the average empirical and model-derived trust matrices, obtaining a value of 0.923. This high correlation confirms that MAR-CCGP produces trustworthiness estimates that quantitatively track observed annotator performance. Figure 11 reinforces this result by illustrating the per-sample trust profiles across clusters, showing coherent spatial trends: Annotators 2 and 3 display localized peaks of competence (e.g., Annotator 3 in Cluster 3), while Annotator 5 exhibits uniformly low trust, consistent with its low SNR settings. Overall, the model delivers interpretable, region-sensitive trust assessments grounded in its inferred uncertainty structure.

**Figure 12:**
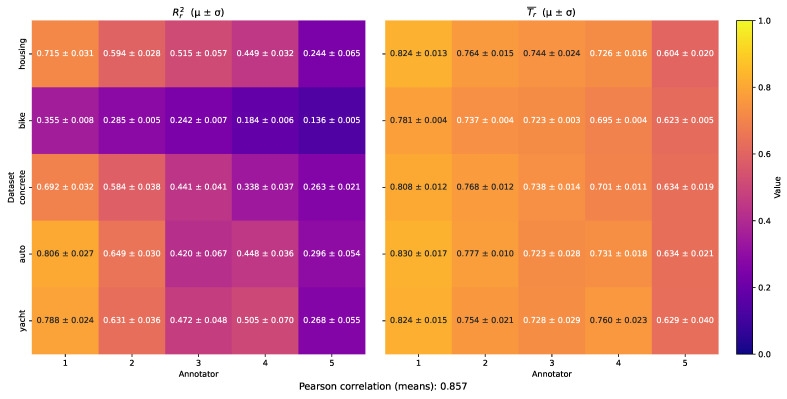
Overall recovery of annotator reliability across all semi-synthetic benchmarks. (**Left**) Mean empirical $R_{r}^{2}$ between each annotator’s noisy labels and known ground truth, averaged over all datasets and repetitions. (**Right**) Corresponding mean MAR-CCGP trustworthiness ${\bar{T}}_{r}$. The strong Pearson correlation (0.853) demonstrates that MAR-CCGP tracks true annotator performance under a variety of noise patterns; small deviations highlight instances where model uncertainty is greatest.

To summarize these findings across all datasets, we computed per-annotator average trustworthiness scores and empirical $R^{2}$ values over input-space clusters, yielding the aggregated heatmaps in Figure 12. The left panel reflects annotator agreement with the uncorrupted ground truth, while the right presents MAR-CCGP’s estimated trustworthiness using only noisy labels. Overall, Annotator 1 consistently achieves the highest empirical $R^{2}$ and estimated trustworthiness scores across datasets, in line with its simulated high and uniform SNR. Conversely, Annotator 5 shows the lowest empirical reliability and is also assigned the lowest trustworthiness by the model. The remaining annotators exhibit dataset-specific patterns, with MAR-CCGP capturing these variations accurately—demonstrating notably strong agreement between empirical and model-derived metrics. For instance, although alignment is generally lower for the Bike Sharing dataset, the corresponding trustworthiness scores are also reduced, reflecting the increased noise and complexity in that domain. Quantitatively, the Pearson correlation between the flattened empirical and model-estimated trustworthiness scores across all datasets reaches 0.857, confirming the model’s capacity to produce reliable and interpretable estimates of annotator behavior based solely on noisy observations.

### 4.2. LUKER-CACAO Dataset Results

Having demonstrated the predictive performance and trustworthiness inference capabilities of MAR-CCGP on semi-synthetic data, we now turn to a real-world application using the LUKER-CACAO dataset described in Section 2.1. Unlike the controlled benchmarks, this sensory evaluation dataset contains separate, curated input–output tables for each of the eight sensory attributes (acidity, bitterness, aroma, astringency, sweetness, hardness, melting speed, and global impression), along with physicochemical features measured per sample (moisture, fat content, granulometry, plastic viscosity, yield stress). Importantly, the dataset contains ratings from five annotators without ground-truth labels, making this an ideal setting for MAR-CCGP to estimate a consensus profile and explore localized annotator trustworthiness.

Following the same experimental setup as the semi-synthetic case (training and testing over 15 random repetitions with 70/30 splits), we applied MAR-CCGP to each sensory variable separately. This setup enables a fair and consistent comparison across attributes and ensures that observed trends reflect annotator behavior rather than sampling variability.

**Figure 13:**
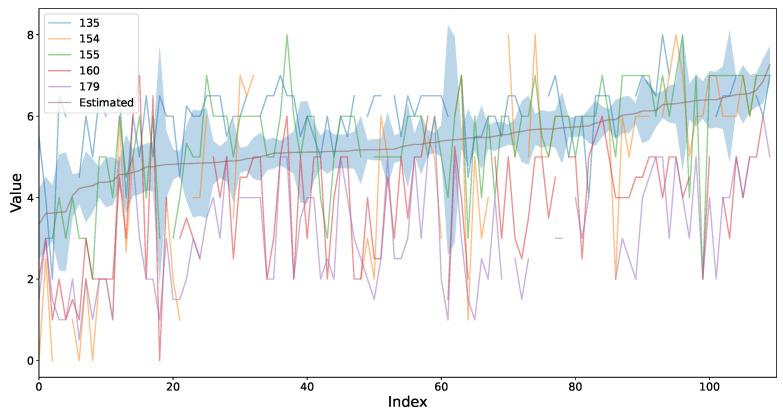
Estimated ground truth sweetness (solid brown line) and its associated predictive uncertainty (shaded region), compared against individual annotators’ sweetness scores for the LUKER-CACAO dataset. Samples are sorted by the model’s estimated mean sweetness value.

Figure 13 shows the estimated mean sweetness profile learned by the model across all samples, along with its 95% predictive credible intervals. Individual annotator ratings are overlaid for direct comparison. The model’s mean estimate lies centrally among the noisy annotator ratings, especially where agreement is high, and predictive intervals widen in the left-hand region of the plot where annotators strongly disagree. Conversely, intervals narrow as ratings converge at higher sweetness levels, demonstrating the model’s ability to represent both consensus and uncertainty appropriately.

**Figure 14:**
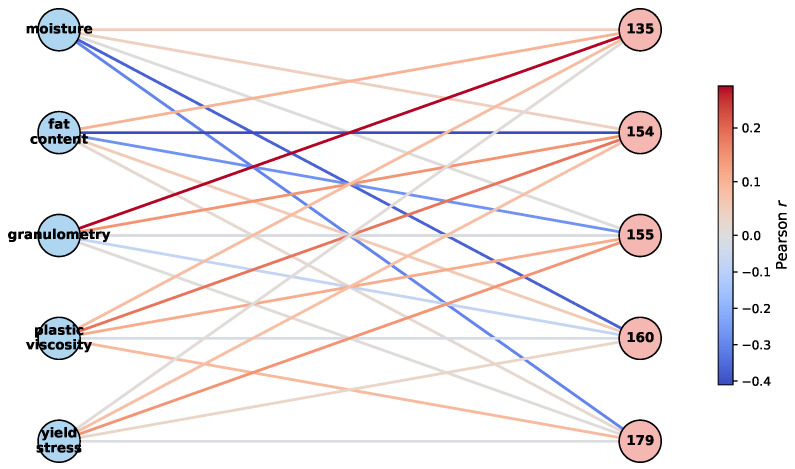
Bipartite graph representing the Pearson correlations between each physicochemical feature (**left**, blue nodes) and the estimated trustworthiness of each annotator (**right**, pink nodes). Edge color encodes the magnitude and sign of the correlation, with red indicating positive and blue indicating negative correlations.

To further explore annotator-specific behavior, we investigated correlations between physicochemical properties and MAR-CCGP-inferred trustworthiness scores. Figure 14 presents a bipartite graph of these relationships. Nodes on the left represent the five physicochemical features, while nodes on the right represent the five annotators. Edge color indicates the Pearson correlation between a feature’s value and an annotator’s estimated trustworthiness across all samples. Warm edges show positive correlations and cool edges show negative correlations. The graph reveals that several annotators—most notably 154 and 160—exhibit negative correlations with *fat content* and *moisture*, suggesting that as these features increase, sweetness ratings become less reliable for these annotators. This is consistent with the intuition that fat and moisture may mask sweetness perception. Conversely, granulometry and yield stress show weaker or even positive correlations for some annotators (e.g., Annotator 135), implying that these panelists evaluate sweetness more reliably under these physical conditions.

**Figure 15:**
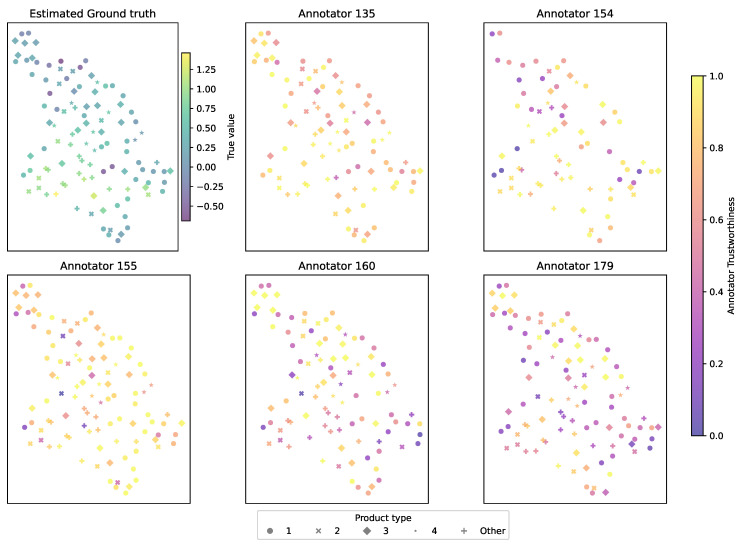
UMAP two-dimensional projection of the LUKER-CACAO dataset for the sweetness attribute. Each point represents a sample, with marker shape indicating product category. (**Top–left panel**) MAR-CCGP’s inferred ground-truth sweetness scores (color shading from low to high). (**Remaining panels**) Annotator-specific trustworthiness scores for annotators 135, 154, 155, 160, and 179 (panels ordered by annotator ID). The UMAP embedding preserves high-dimensional neighborhood structure, allowing the reader to see how both predicted sweetness and annotator confidence vary across different regions of the physicochemical space.

To visualize trustworthiness across the physicochemical input space, Figure 15 depicts UMAP projections of the cacao samples, color-coded by MAR-CCGP’s estimated trustworthiness per annotator. The top-left panel depicts the estimated mean sweetness profile projected into the UMAP space, serving as a baseline. The remaining panels show each annotator’s trust scores across this space. Annotators 154 and 155 achieve high trustworthiness across most regions (consistently warm yellow hues), indicating stable and reliable performance. Annotators 160 and 179, by contrast, display region-specific drops in trustworthiness, especially for clusters of samples with high moisture or fat content. These localized patterns highlight systematic variations in annotator behavior across products.

**Figure 16:**
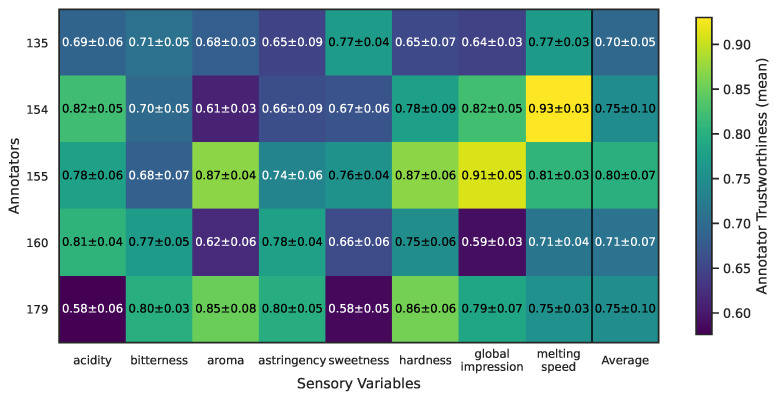
Summary heatmap of average annotator trustworthiness (μ±σ) across all sensory variables and annotators, showing per-attribute trustworthiness profiles derived by MAR-CCGP. Values represent the mean trust scores with their corresponding standard deviations, and color indicates the relative trust level.

Finally, Table 7 and Figure 16 provide a detailed summary of estimated annotator trustworthiness across all sensory variables and product types. Consistent with the qualitative UMAP patterns, Annotators 154 and 155 stand out as the most consistently trustworthy panelists, yielding mean trust scores above 0.80 for most attributes with low standard deviations across different product types. This is evident not only in sweetness but also for aroma, melting speed, hardness, and global impression, where both annotators maintain strong and stable reliability. Annotator 160, by contrast, exhibits markedly lower mean trustworthiness across many attributes (typically in the 0.60–0.70 range), with larger standard deviations—especially for perceptually challenging attributes like astringency and bitterness—indicating highly variable performance that depends on the product’s physicochemical profile. Annotator 179 also shows a similar pattern of reduced consistency, attaining good trust scores for a few localized product types but generally underperforming relative to the most consistent annotators. Interestingly, some sensory variables, such as bitterness and astringency, present inherently greater variability in trust scores across all panelists, suggesting that these attributes may be more difficult to evaluate reliably across the diverse product set. In contrast, more mechanically defined properties like hardness and melting speed exhibit higher and more uniform trustworthiness estimates across all annotators, which could reflect the greater perceptual salience of these sensory dimensions. Statistical evaluation supports these observations: Friedman omnibus tests run for each variable–product block confirm significant differences among annotators (all *p* < 0.05 except a few non-significant cases), and Nemenyi post-hoc comparisons consistently place Annotators 154 and 155 in the top performance group, while Annotators 160 and 179 fall into lower-ranked groups. Taken together, these findings demonstrate that MAR-CCGP successfully disentangles systematic annotator-specific behavior from product-specific effects, yielding granular trustworthiness estimates that highlight where particular panelists excel or struggle. This level of insight can inform sensory panel calibration, personalized training, and targeted quality control by drawing attention to specific attributes and product types that require additional guidance or closer monitoring.

**Figure 17:**
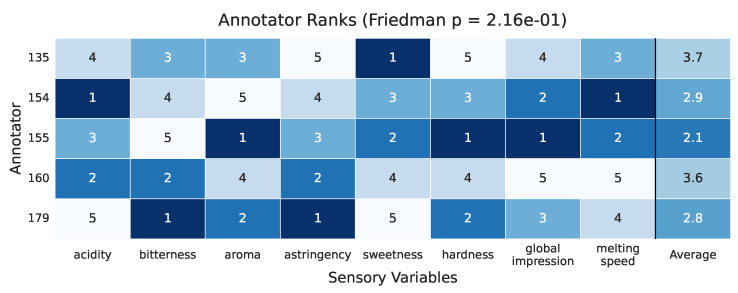
Annotator-wise ranks across sensory variables (lower is better), derived from the Friedman test analysis. The Friedman *p*-value indicates no statistically significant differences in global rank (p=0.216), but relative ranks help interpret performance trends across variables.

To complement the absolute trust score comparisons, we conducted a non-parametric Friedman test to assess whether annotator performance differs significantly across sensory variables. For this analysis, the mean trust score per annotator was computed for each variable, and the annotators were ranked accordingly. These ranks were used as inputs to the Friedman test, which yielded a non-significant result (p=0.216), suggesting that no annotator consistently outperforms others across *all* sensory dimensions. Nonetheless, the annotator-wise rank profiles, visualized in Figure 17, provide meaningful insights into relative strengths. Annotator 3 attains the **lowest average rank (2.1)**, indicating consistently strong performance across variables like aroma, hardness, and global impression. In contrast, Annotator 1 obtains the highest mean rank (3.7), reflecting comparatively lower performance. These results offer an interpretable complement to raw trust scores and reinforce that while some annotators excel on specific sensory attributes, differences are not uniformly significant across the entire panel.

Together, these findings demonstrate MAR-CCGP’s capacity to extract nuanced, input-dependent trustworthiness estimates even in real-world sensory settings without ground-truth supervision. The model’s trust scores closely align with expected behavior derived from physicochemical features, and its uncertainty estimates highlight regions where panel agreement is weakest. Importantly, these insights offer actionable guidance for panelist training and calibration, allowing sensory scientists to identify panelists who struggle with specific product types and potentially target these areas for further calibration or retraining.

### 4.3. Limitations

While the MAR-CCGP framework provides a robust solution for integrating multi-annotator sensory data with physicochemical profiles, several limitations remain. First, the model assumes that annotator reliability can be effectively captured through input-dependent variance, which may oversimplify annotators’ behavior in scenarios involving complex biases or strategic labeling. Second, the proposed trustworthiness score relies on probabilistic estimations derived from model outputs, but its interpretation may be challenging in the absence of external validation mechanisms or behavioral ground truth. Moreover, although the method performs well in semi-synthetic and real-world settings, it is currently limited to low-dimensional input spaces with a manageable number of annotators; scalability to high-dimensional sensory domains or crowd-scale scenarios may require further optimization or approximation strategies. Additionally, the framework depends on Gaussian process inference, which incurs high computational costs as the dataset size increases. Finally, while the LUKER-CACAO dataset enables a valuable real-world demonstration, its proprietary nature restricts reproducibility and broader benchmarking by the community.

## 5. Conclusions

We introduce a Multi-Annotator Regression framework based on Correlated Chained Gaussian Processes, named MAR-CCGP, a novel multi-annotator approach that jointly models continuous sensory scores and input-dependent annotator reliability through a probabilistic Gaussian process formulation. The key conceptual innovation lies in disentangling true perceptual signals from annotator-specific noise using a latent consensus function and localized trust estimation. Unlike previous methods that assume uniform or global annotator performance, MAR-CCGP learns region-specific annotator trustworthiness scores, enabling interpretability and robustness in the face of sparse, noisy, and subjective supervision. The model is particularly suited for domains like food science, where expert annotations are limited and often exhibit contextual biases.

Our results demonstrate the effectiveness of MAR-CCGP across both real-world and semi-synthetic settings. On the proprietary LUKER-CACAO dataset, the model achieved strong predictive performance for multiple sensory attributes and provided meaningful trust scores that aligned with empirical annotator behavior, especially in dimensions such as bitterness and aroma. In controlled experiments with structured SNR profiles, MAR-CCGP outperformed consensus-only and local-weighting baselines in both RMSE and reliability estimation. Importantly, the model’s ability to recover annotator-specific patterns in low-trust clusters highlights its utility in curating more reliable datasets and guiding future annotation efforts. These findings suggest that MAR-CCGP not only enhances regression accuracy under label noise but also supports informed decision-making through interpretable reliability scores. Overall, MAR-CCGP offers a suitable and interpretable solution for learning from subjective, sparse, and inconsistent annotations, with significant implications for robust modeling in food quality control. To the best of our knowledge, this is the first work to integrate a curated sensory–physicochemical cacao dataset, regression under noisy and scarce annotations, and input-dependent annotator trust estimation in a single probabilistic framework that naturally quantifies uncertainty and adapts to varying data quality.

Future research could explore scaling MAR-CCGP to high-dimensional input domains and larger annotator pools by integrating sparse approximations or deep kernel learning methods [51,52]. Another promising direction involves extending the framework to heteroscedastic multi-output settings, allowing the simultaneous modeling of correlations between multiple sensory targets and annotator behaviors. Incorporating behavioral signals or auxiliary metadata from annotators—such as labeling time, confidence, or experience level—could further refine trust estimation [53,54]. Lastly, validating the trust scores through longitudinal studies or expert-in-the-loop experiments may reinforce their adoption in high-stakes domains such as medical diagnostics, environmental monitoring, and consumer preference modeling.

## Figures and Tables

**Table 1:** Standard analytical methods used for each selected physicochemical and sensory variables.

Variable	Analytical Method
Fat Content	AOAC Official Method 963.15 [38]
Moisture	AOAC Official Method 931.04 [39]
Granulometry	ISO 13320:2020 [40]
Plastic Viscosity	IOCCC Method 46 [41]
Yield Stress	IOCCC Method 46 [41]
Sensory Attributes	NTC 3932 [42]

**Table 2:** Label completeness (%) per annotator and sensory variable in the cacao-based product database. Each value represents the percentage of non-missing labels relative to the total number of retained samples for that attribute.

Annotator	Acidity	Bitterness	Aroma	Astringency	Sweetness	Hardness	Global Impression	Melting Speed
135	86.1	86.1	85.6	90.2	85.5	85.5	85.5	85.5
154	70.8	70.8	69.4	68.9	69.1	69.1	69.1	69.1
155	95.8	95.8	97.3	95.1	97.3	97.3	97.3	97.3
160	88.9	88.9	90.1	90.2	90.0	90.0	90.0	90.0
179	88.9	88.9	91.9	91.8	91.8	91.8	91.8	91.8
Available samples	72	72	111	61	110	110	110	110

**Table 3:** Completeness (%) of input features and number of samples per task for the Cacao-based product database. Completeness is measured as the percentage of non-missing values for each physicochemical variable in the final dataset associated with each sensory attribute.

Physicochemical/ Sensory	Acidity	Bitterness	Aroma	Astringency	Sweetness	Hardness	Global Impression	Melting Speed
Moisture	98.6	98.6	99.1	98.4	99.1	99.1	99.1	99.1
Fat Content	95.8	95.8	97.3	95.1	97.3	97.3	97.3	97.3
Granulometry	88.9	88.9	91.9	86.9	92.7	92.7	92.7	92.7
Plastic viscosity	100.0	100.0	100.0	100.0	100.0	100.0	100.0	100.0
Yield stress	97.2	97.2	98.2	96.7	98.2	98.2	98.2	98.2
Available samples	72	72	111	61	110	110	110	110

**Table 4:** Summary of benchmark regression datasets from the UCI repository used for simulating semi-synthetic multi-annotator annotations.

Dataset	# Samples	# Features
Bike Sharing	17,379	11
Concrete Strength	1030	8
Boston Housing	501	13
Auto MPG	392	7
Yacht Hydrodynamics	308	6

**Table 5:** Comparative summary of baseline and advanced models for multi-annotator regression.

Method	Acronym	Description
Gaussian Process on Ground Truth [30]	GPR-GT	Supervised GP regression trained on true outputs. Serves as an oracle upper bound and assumes full access to ground truth. Does not model annotators or uncertainty.
Gaussian Process on Average Annotations [23]	GPR-AVG	GP trained on the per-instance average of annotator targets. Assumes annotators are unbiased and neglects individual reliability. Serves as a baseline that models only the consensus.
Localized Kernel Alignment-based Annotator Relevance [31]	LKAAR	Jointly estimates annotator bias and variance, and embeds annotator consistency as a kernelized function over the input space. Provides localized reliability estimates.
Multi-Annotator Regression based on Correlated Chained Gaussian Process (ours)	MAR-CCGP	Proposed model. Captures latent inter-annotator correlations and input-dependent noise via correlated-chained latent functions and sparse variational GPs. Produces localized consistency-trustworthiness estimates.

**Table 6:** Global regression measures for the semi-synthetic datasets. Values are mean ± standard deviation across 15 folds for each method and dataset. Abbreviations: Mean Squared Error (MSE); Mean Absolute Error (MAE); Mean Absolute Percentage Error (MAPE); Coefficient of Determination ($R^{2}$). Bold values indicate the best performance for each metric.

Dataset	Method	MSE	MAE	MAPE	$R^{2}$
Boston Housing	GPR-AVG	0.247±0.066	0.321±0.026	1.947±0.543	0.763±0.047
	GPR-GT	0.191±0.043	0.286±0.016	1.899±0.504	0.815±0.031
	LKAAR	0.313±0.080	0.358±0.033	2.023±0.516	0.700±0.051
	MAR-CCGP	0.189±0.053	0.279±0.022	1.670±0.452	0.818±0.040
Bike Sharing	GPR-AVG	0.690±0.015	0.623±0.017	2.122±0.132	0.315±0.008
	GPR-GT	0.678±0.011	0.616±0.016	2.224±0.141	0.327±0.007
	LKAAR	0.720±0.012	0.641±0.008	1.890±0.116	0.285±0.004
	MAR-CCGP	0.614±0.013	0.558±0.013	2.319±0.131	0.390±0.009
Concrete Strength	GPR-AVG	0.325±0.024	0.452±0.016	1.869±0.331	0.676±0.029
	GPR-GT	0.273±0.023	0.411±0.015	1.660±0.279	0.728±0.026
	LKAAR	0.384±0.027	0.499±0.018	1.799±0.344	0.617±0.023
	MAR-CCGP	0.227±0.022	0.367±0.015	1.568±0.295	0.773±0.028
Auto MPG	GPR-AVG	0.154±0.024	0.280±0.022	0.919±0.264	0.848±0.018
	GPR-GT	0.135±0.017	0.263±0.018	0.841±0.237	0.867±0.015
	LKAAR	0.184±0.031	0.304±0.028	0.921±0.243	0.819±0.020
	MAR-CCGP	0.126±0.018	0.255±0.017	0.817±0.241	0.875±0.018
Yacht Hydrodynamics	GPR-AVG	0.169±0.044	0.295±0.036	0.843±0.148	0.832±0.023
	GPR-GT	0.095±0.021	0.226±0.016	0.702±0.157	0.904±0.009
	LKAAR	0.262±0.066	0.354±0.043	0.962±0.167	0.739±0.030
	MAR-CCGP	0.105±0.036	0.208±0.028	0.633±0.186	0.896±0.020

**Table 7:** Annotator trustworthiness (Mean ± Std) and statistical comparison for each variable and product. Column “Friedman” gives $\left(\chi^{2},p\right)$; superscripts denote Nemenyi groups (same letter = not significantly different at α=0.05). Bold values indicate the best performance for each metric.

Variable	Prod	Friedman ($\chi^{2}$, *p*)	135	154	155	160	179
acidity	1	42.35, 0.000	0.63 ± 0.09 ^*b*^	**0.79 ± 0.06** ^*a*^	0.74 ± 0.08 ^*ab*^	0.78 ± 0.07 ^*a*^	0.53 ± 0.08 ^*c*^
	2	12.67, 0.013	0.82 ± 0.10 ^*bc*^	0.89 ± 0.08 ^*ab*^	0.88 ± 0.08 ^*ab*^	**0.90 ± 0.05** ^*a*^	0.79 ± 0.08 ^*bc*^
	3	24.33, 0.000	0.85 ± 0.14 ^*ab*^	0.79 ± 0.10 ^*bc*^	**0.88 ± 0.11** ^*a*^	0.74 ± 0.07 ^*c*^	0.83 ± 0.14 ^*ab*^
	4	9.37, 0.052	0.70 ± 0.20 ^*c*^	**0.86 ± 0.09** ^*a*^	0.72 ± 0.17 ^*c*^	0.83 ± 0.07 ^*ab*^	0.53 ± 0.12 ^*d*^
	General	43.73, 0.000	0.80 ± 0.19 ^*b*^	**0.88 ± 0.10** ^*a*^	0.84 ± 0.18 ^*ab*^	0.88 ± 0.09 ^*a*^	0.69 ± 0.20 ^*c*^
bitterness	1	31.04, 0.000	0.70 ± 0.06 ^*bc*^	0.68 ± 0.06 ^*c*^	0.67 ± 0.08 ^*c*^	**0.84 ± 0.06** ^*a*^	0.83 ± 0.04 ^*ab*^
	2	22.67, 0.000	0.59 ± 0.19 ^*b*^	0.47 ± 0.24 ^*c*^	0.56 ± 0.18 ^*b*^	0.48 ± 0.24 ^*c*^	**0.67 ± 0.11** ^*a*^
	3	10.47, 0.033	0.93 ± 0.04 ^*a*^	**0.99 ± 0.01** ^*a*^	0.85 ± 0.12 ^*b*^	0.52 ± 0.18 ^*c*^	0.53 ± 0.27 ^*c*^
	4	3.66, 0.454	0.73 ± 0.14 ^*ab*^	0.79 ± 0.19 ^*a*^	0.71 ± 0.19 ^*ab*^	0.71 ± 0.13 ^*ab*^	**0.90 ± 0.06** ^*a*^
	General	22.13, 0.000	0.75 ± 0.19 ^*b*^	**0.77 ± 0.27** ^*a*^	0.70 ± 0.23 ^*bc*^	0.62 ± 0.25 ^*c*^	0.66 ± 0.27 ^*bc*^
aroma	1	48.75, 0.000	0.65 ± 0.05 ^*b*^	0.60 ± 0.05 ^*c*^	**0.92 ± 0.04** ^*a*^	0.69 ± 0.07 ^*b*^	0.84 ± 0.09 ^*ab*^
	2	25.79, 0.000	0.63 ± 0.06 ^*bc*^	0.63 ± 0.12 ^*bc*^	0.79 ± 0.13 ^*b*^	0.65 ± 0.23 ^*bc*^	**0.84 ± 0.18** ^*ab*^
	3	42.24, 0.000	0.71 ± 0.06 ^*b*^	0.51 ± 0.12 ^*c*^	0.79 ± 0.06 ^*b*^	0.52 ± 0.14 ^*c*^	**0.84 ± 0.12** ^*ab*^
	4	26.31, 0.000	0.83 ± 0.14 ^*a*^	0.72 ± 0.01 ^*b*^	**0.90 ± 0.17** ^*a*^	0.29 ± 0.12 ^*c*^	0.73 ± 0.26 ^*b*^
	General	37.47, 0.000	0.73 ± 0.17 ^*b*^	0.66 ± 0.13 ^*c*^	**0.86 ± 0.14** ^*a*^	0.59 ± 0.25 ^*bc*^	0.86 ± 0.18 ^*a*^
astringency	1	33.87, 0.000	0.65 ± 0.09 ^*bc*^	0.70 ± 0.08 ^*b*^	0.72 ± 0.06 ^*b*^	0.80 ± 0.06 ^*ab*^	**0.84 ± 0.05** ^*a*^
	2	30.10, 0.000	0.62 ± 0.09 ^*b*^	0.56 ± 0.23 ^*c*^	0.70 ± 0.24 ^*b*^	0.46 ± 0.17 ^*c*^	**0.85 ± 0.17** ^*a*^
	3	16.00, 0.003	0.52 ± 0.11 ^*c*^	0.46 ± 0.11 ^*d*^	**0.90 ± 0.06** ^*a*^	0.83 ± 0.10 ^*b*^	0.97 ± 0.02 ^*a*^
	4	6.40, 0.171	0.71 ± 0.12 ^*ab*^	0.46 ± 0.14 ^*c*^	**0.81 ± 0.15** ^*a*^	0.79 ± 0.10 ^*ab*^	0.63 ± 0.20 ^*b*^
	General	27.52, 0.000	0.65 ± 0.15 ^*bc*^	0.53 ± 0.16 ^*d*^	**0.77 ± 0.18** ^*a*^	0.73 ± 0.20 ^*b*^	0.76 ± 0.28 ^*b*^
sweetness	1	49.33, 0.000	0.80 ± 0.05 ^*a*^	0.55 ± 0.11 ^*c*^	**0.82 ± 0.06** ^*a*^	0.52 ± 0.07 ^*c*^	0.43 ± 0.06 ^*d*^
	2	32.58, 0.000	0.72 ± 0.11 ^*b*^	0.61 ± 0.15 ^*c*^	0.52 ± 0.18 ^*d*^	0.70 ± 0.11 ^*bc*^	**0.77 ± 0.15** ^*a*^
	3	33.07, 0.000	0.73 ± 0.08 ^*bc*^	0.79 ± 0.12 ^*b*^	0.74 ± 0.06 ^*bc*^	**0.93 ± 0.07** ^*a*^	0.84 ± 0.08 ^*ab*^
	4	19.00, 0.001	0.84 ± 0.13 ^*a*^	**0.88 ± 0.05** ^*a*^	0.80 ± 0.13 ^*ab*^	0.62 ± 0.21 ^*c*^	0.52 ± 0.11 ^*c*^
	General	45.92, 0.000	0.76 ± 0.17 ^*bc*^	**0.80 ± 0.16** ^*a*^	0.72 ± 0.19 ^*c*^	0.69 ± 0.19 ^*c*^	0.66 ± 0.21 ^*c*^
hardness	1	39.73, 0.000	0.61 ± 0.08 ^*c*^	0.87 ± 0.08 ^*b*^	0.87 ± 0.06 ^*b*^	0.73 ± 0.10 ^*c*^	**0.88 ± 0.05** ^*a*^
	2	17.59, 0.001	0.62 ± 0.09 ^*c*^	0.85 ± 0.12 ^*b*^	0.84 ± 0.16 ^*b*^	0.77 ± 0.12 ^*bc*^	**0.89 ± 0.14** ^*a*^
	3	19.57, 0.001	0.67 ± 0.12 ^*c*^	0.77 ± 0.26 ^*bc*^	0.89 ± 0.07 ^*b*^	0.88 ± 0.09 ^*b*^	**0.90 ± 0.07** ^*a*^
	4	18.00, 0.001	0.70 ± 0.10 ^*c*^	0.87 ± 0.09 ^*b*^	**0.94 ± 0.08** ^*a*^	0.73 ± 0.12 ^*c*^	0.86 ± 0.10 ^*b*^
	General	38.13, 0.000	0.67 ± 0.21 ^*c*^	0.59 ± 0.34 ^*d*^	**0.84 ± 0.21** ^*a*^	0.70 ± 0.26 ^*bc*^	0.82 ± 0.22 ^*b*^
global impression	1	41.17, 0.000	0.69 ± 0.06 ^*b*^	0.85 ± 0.08 ^*ab*^	**0.91 ± 0.06** ^*a*^	0.64 ± 0.06 ^*b*^	0.77 ± 0.09 ^*ab*^
	2	22.98, 0.000	0.55 ± 0.07 ^*c*^	0.75 ± 0.20 ^*b*^	**0.96 ± 0.07** ^*a*^	0.55 ± 0.11 ^*c*^	0.83 ± 0.16 ^*b*^
	3	34.77, 0.000	0.65 ± 0.08 ^*b*^	0.81 ± 0.17 ^*ab*^	**0.88 ± 0.11** ^*a*^	0.52 ± 0.10 ^*c*^	0.86 ± 0.11 ^*ab*^
	4	25.60, 0.000	0.39 ± 0.11 ^*d*^	0.96 ± 0.08 ^*a*^	**0.98 ± 0.02** ^*a*^	0.42 ± 0.20 ^*d*^	0.50 ± 0.28 ^*cd*^
	General	53.49, 0.000	0.57 ± 0.21 ^*c*^	0.82 ± 0.16 ^*b*^	**0.91 ± 0.18** ^*a*^	0.58 ± 0.15 ^*c*^	0.78 ± 0.25 ^*b*^
melting speed	1	36.96, 0.000	0.77 ± 0.05 ^*bc*^	**0.97 ± 0.05** ^*a*^	0.80 ± 0.05 ^*b*^	0.75 ± 0.06 ^*c*^	0.73 ± 0.04 ^*c*^
	2	20.66, 0.000	0.88 ± 0.07 ^*a*^	0.89 ± 0.17 ^*a*^	0.89 ± 0.04 ^*a*^	0.61 ± 0.17 ^*c*^	0.77 ± 0.02 ^*ab*^
	3	36.00, 0.000	0.77 ± 0.07 ^*c*^	0.98 ± 0.03 ^*ab*^	0.75 ± 0.08 ^*c*^	0.72 ± 0.09 ^*c*^	**0.78 ± 0.03** ^*a*^
	4	28.30, 0.000	0.90 ± 0.08 ^*b*^	**1.00 ± 0.01** ^*a*^	0.94 ± 0.06 ^*ab*^	0.64 ± 0.10 ^*c*^	0.72 ± 0.03 ^*bc*^
	General	46.77, 0.000	0.77 ± 0.19 ^*bc*^	0.78 ± 0.27 ^*bc*^	**0.81 ± 0.11** ^*a*^	0.68 ± 0.21 ^*c*^	0.77 ± 0.06 ^*bc*^

## Data Availability

Semi-synthetics datasets are publicly available at: https://github.com/UN-GCPDS/python-gcpds.luker_multiple_annotators (accessed on 1 April 2025). Access to the LUKER-CACAO dataset is restricted due to copyright limitations.
